# *MPPED2* Polymorphism Is Associated With Altered Systemic Inflammation and Adverse Trauma Outcomes

**DOI:** 10.3389/fgene.2019.01115

**Published:** 2019-11-08

**Authors:** Lukas Schimunek, Rami A. Namas, Jinling Yin, Derek Barclay, Dongmei Liu, Fayten el-Dehaibi, Andrew Abboud, Maria Cohen, Ruben Zamora, Timothy R. Billiar, Yoram Vodovotz

**Affiliations:** ^1^Department of Surgery, University of Pittsburgh, Pittsburgh, PA, United States; ^2^Department of Anesthesiology, University of Pittsburgh, Pittsburgh, PA, United States; ^3^Center for Inflammation and Regeneration Modeling, McGowan Institute for Regenerative Medicine, University of Pittsburgh, Pittsburgh, PA, United States

**Keywords:** inflammation, SNP, trauma, systems biology, genomics, outcomes, ventilation

## Abstract

Trauma is a leading cause of morbidity and mortality. It is unclear why some trauma victims follow a complicated clinical course and die, while others, with apparently similar injury characteristics, do not. Interpatient genomic differences, in the form of single nucleotide polymorphisms (SNPs), have been associated previously with adverse outcomes after trauma. Recently, we identified seven novel SNPs associated with mortality following trauma. The aim of the present study was to determine if one or more of these SNPs was also associated with worse clinical outcomes and altered inflammatory trajectories in trauma survivors. Accordingly, of 413 trauma survivors, DNA samples, full blood samples, and clinical data were collected at multiple time points in the first 24 h and then daily over 7 days following hospital admission. Subsequently, single-SNP groups were created and outcomes, such as hospital length of stay (LOS), ICU LOS, and requirement for mechanical ventilation, were compared. Across a broad range of Injury Severity Scores (ISS), patients carrying the rs2065418 TT SNP in the metallophosphoesterase domain-containing 2 (*MPPED2*) gene exhibited higher Marshall MODScores vs. the control group of rs2065418 TG/GG patients. In patients with high-severity trauma (ISS ≥ 25, *n* = 94), those carrying the rs2065418 TT SNP in *MPPED2* exhibited higher Marshall MODScores, longer hospital LOS (21.8 ± 2 days), a greater requirement for mechanical ventilation (9.2 ± 1.4 days on ventilator, DOV), and higher creatinine plasma levels over 7 days vs. the control group of rs2065418 TG/GG high-severity trauma patients (LOS: 15.9 ± 1.2 days, *p* = 0.03; DOV: 5.7 ± 1 days, *p* = 0.04; plasma creatinine; *p* < 0.0001 MODScore: *p* = 0.0003). Furthermore, rs2065418 TT patients with ISS ≥ 25 had significantly different plasma levels of nine circulating inflammatory mediators and elevated dynamic network complexity. These studies suggest that the rs2065418 TT genotype in the *MPPED2* gene is associated with altered systemic inflammation, increased organ dysfunction, and greater hospital resource utilization. A screening for this specific SNP at admission might stratify severely injured patients regarding their lung and kidney function and clinical complications.

## Introduction

Traumatic injury is the third leading cause of death in the United States, with estimated costs exceeding $130 billion annually (Palmer, 2007; Soreide, 2009; Dwyer-Lindgren et al., 2016). Injury severity is commonly evaluated using the Injury Severity Score (ISS) derived from the Abbreviated Injury Scale (AIS) (Baker et al., 1974). Major trauma resulting in an ISS ≥ 16 is considered “moderate,” while an ISS ≥ 25 is considered “severe” trauma (Palmer, 2007; Almahmoud et al., 2015). ISS is positively correlated with altered systemic inflammation, increasing rates of complications such as multiple organ dysfunction (MOD, which can be evaluated using the Marshall MODScore among others), prolonged hospitalization, and increased mortality (Pettit et al., 2014; Almahmoud et al., 2015). Additionally, survivors of major trauma suffer from a reduced quality of life even years after trauma (Michaels et al., 2000; Sluys et al., 2005; Ringdal et al., 2009).

Studies on the human genome have led to the hypothesis that genetic variability, especially of genes involved in the inflammatory response, can contribute to outcomes following severe traumatic injury (Hildebrand et al., 2011; Bronkhorst et al., 2015). Genetic variability often occurs in the form of single nucleotide polymorphisms (SNPs). SNPs are defined as single variants of one base in the genome and are the most common type of inter-individual genetic variability (Syvanen, 2001).

Numerous studies have suggested that candidate SNPs—predominantly in genes encoding inflammatory cytokines (Heesen et al., 2002; Majetschak et al., 2002; Duan et al., 2011; Jeremic et al., 2014), their receptors (Thompson et al., 2014), and downstream signaling cascades (Sperry et al., 2014)—are associated with adverse outcomes following trauma. In a recent study, we screened for trauma outcome-related SNPs, identifying a set of seven SNP genotypes in six different genes (from a total of 551,839 screened SNPs) which, together, were associated with an altered inflammatory response and non-survival (Schimunek et al., 2018). Surprisingly, none of the genes containing these SNPs was involved directly in any known inflammatory pathway. Rather, the genes were related to basic tissue metabolism and function. For three of these seven SNPs, neither the genes nor their products are known. For the remaining four SNPs, more information is available. Reference (Ref) SNPs rs2241777 and rs3134287 are located in the gene encoding for the protein solute carrier family 25 member 32 (SLC25A32), which is involved in the transportation of flavin adenine dinucleotide (FAD) over the mitochondrial membrane, ultimately helping drive the production of adenosine triphosphate (ATP) (Spaan et al., 2005). Ref SNP rs3098223 is located in the gene encoding for DDB1- and CUL4-associated factor 13 (DCAF13). DCAFs are substrates of the CUL4-DDB1 ubiquitin ligase, which is a regulator of cell proliferation and survival (Lee and Zhou, 2007). Ref SNP rs2065418 lies in the gene encoding for metallophosphoesterase domain-containing protein 2 (*MPPED2*). *MPPED2* is associated with cell proliferation (Liguori et al., 2012), lung injury after mechanical ventilation (Kompass et al., 2010), and kidney function (Pattaro et al., 2012; Schimunek et al., 2018).

Herein, we sought to extend our prior studies in order to determine if there was a single SNP among these seven SNPs that could stratify clinical outcomes and inflammatory trajectories of trauma survivors in the same population of 413 blunt trauma survivors in which we discovered the seven SNPs. Subsequently, we aimed to define its association individually with dynamic changes in systemic inflammation as well as with clinical outcomes following severe blunt trauma.

## Materials and Methods

### Patients

Four hundred thirteen blunt trauma survivors [mean age: 49.5 ± 1 years (min: 18 years, max: 90 years); mean ISS: 19.2 ± 0.5 (min: 1, max: 54); gender: 130 females, 283 males; ethnicity: 386 Caucasians, 11 African-Americans, 4 Asians, 12 unknown] were enrolled for initial screening, after admission to the emergency department of the UPMC Presbyterian hospital (a level 1 trauma center), following Institutional Review Board approval and obtaining informed consent. DNA samples were obtained upon admission to the trauma bay. Clinical and biochemical data were collected from the electronic medical records. The primary focus of this study was on the association of potential SNPs following severe injury. Accordingly, we focused on the 94 severely injured (ISS ≥ 25) blunt trauma survivors of the 413 blunt trauma survivors [mean age: 42.3 ± 2 years (min: 18 years, max: 89 years); mean ISS: 34.3 ± 0.8 (min: 25, max: 54); gender: 26 females, 68 males; ethnicity: 87 Caucasians, 3 African-Americans, 2 Asians, 2 unknown].

### Individual SNPs Associated With Trauma Non-Survivors

In a previous study, we associated a set of seven SNPs with trauma non-survival (Schimunek et al., 2018). Of these, three SNPs are located on chromosome 11: rs10741668 [chromosome (Chr) 11; telomere p14.1; position (pos) 15,277,383; base A/G; minor allele frequency (MAF) G = 0.29], Ref SNP rs10790334 (Chr 11; q14; pos 98,895,933; base T/C; MAF C = 0.18), and Ref SNP rs2065418 (Chr 11; p14.1; pos 30,400,521; T/G; MAF G = 0.27). Ref SNP rs2065418 is located inside the *MPPED2* gene, which encodes for metallophosphoesterase domain-containing protein 2. The other two SNPs on chromosome 11 are unknown in both name and function (**Supplementary Table 1**). Another three SNPs are located on chromosome 8: Ref SNP rs2241777 (Chr 8; q22.3; pos 103400160; A/C; MAF A = 0.43), Ref SNP rs3098223 (Chr 8; q22.3; pos 103,434,877; A/G; MAF G = 0.48), and Ref SNP rs3134287 (Chr 8; q22.3; pos 103,411,258; T/C; MAF C = 0.48). Ref SNP rs3098223 is located inside the *DCAF13* gene, coding for DDB1- and CUL4-associated factor 13. Ref SNP rs2241777 and Ref SNP rs3134287 are both located in the *SLC25A32* gene, coding for Solute carrier family 25 member 32. Ref SNP rs906790 is located on chromosome 13 (Chr 13; q21; pos 76,161,264; A/G) and is unknown in both name and function.

### DNA Sampling and Single-Nucleotide Polymorphism Genotyping

DNA was prepared from whole blood samples and analyzed using Illumina^®^ arrays as described previously (Schimunek et al., 2018) and detailed in the **Supplementary Methods**. To validate the Illumina-derived *MPPED2* genotype, real-time polymerase chain reaction (PCR) was carried out as described in the **Supplementary Methods**.

### Analysis of Linkage Disequilibrium

We tested the previously discovered set of seven SNPs for linkage disequilibrium (LD) using online analysis tools provided by NIH, *LDmatrix* for graphics and *LDpair* for statistics (Kim et al., 1989; Machiela and Chanock, 2015). Results are based on all populations of the *Phase 3* of the *1000 Genomes Project* (Genomes Project et al., 2015) and given in both *D*′ and *R* ^2^.

### Serial Analysis of Inflammatory Mediators

Plasma levels of the following 31 inflammatory mediators were analyzed as described previously (Schimunek et al., 2018) and detailed in the *Supplementary Methods*: Eotaxin, GM-CSF, IFN-α, IFN-γ, IL-1β, IL-1RA, IL-2, sIL-2Rα, IL-4, IL-5, IL-6, IL-7, IL-8, IL-9, IL-10, IL-13, IL-15, IL-17A, IL-17E/25, IL-21, IL-22, IL-23, IL-33, IP-10, MCP-1, MIG, MIP-1α, MIP-1β, NO_2_^−^/NO_3_^−^, soluble ST2 (sST2), and TNF-α.

### Statistical Analyses

All analyses were carried out using GraphPad Prism 7 (GraphPad Software, Inc., San Diego, CA). A *p* value of less than 0.05 was considered significant for clinical outcomes; a *p* value of less than 0.01 was considered significant for serial analysis of inflammatory mediators. D’Agostino–Pearson normality test was used to identify if the patient demographics and outcomes were distributed normally. Student’s *t* test was used to compare differences between groups of patients with regard to normally distributed demographics and outcomes. The Mann–Whitney *U* test was used to compare differences between groups of patients with regard to non-normally distributed patient demographics and outcomes. One-Way ANOVA was used for multiple group comparisons regarding normally distributed data, followed by Tukey’s multiple comparisons *post hoc* test. Multiple group comparisons of non-normally distributed data were performed using Kruskal–Wallis test, followed by Dunn’s multiple comparisons *post hoc* test. Fisher’s exact test was used to compare patient demographics and outcomes organized in contingency tables. Two-Way ANOVA was used to determine time-dependent changes of plasma creatinine levels, MODScores, and circulating inflammatory mediators as a function of patient subgroup.

### Dynamic Network Analysis

Dynamic network analysis (DyNA), carried out in Matlab^®^ software, was used to define the central inflammatory network mediators as a function of both time and patient subgroup as described previously (Mi et al., 2011) and further described in the **Supplementary Methods**. Network complexity scores (NCS) were calculated and presented as trajectories. Heat maps were created to show the connectivity of the different inflammatory mediators over time.

## Results

### Analysis of Linkage Disequilibrium of Individual SNPs Associated With Trauma Non-Survivors

We first sought to determine if any of the seven non-survivor-associated SNP genotypes (Schimunek et al., 2018) we associated previously with trauma non-survivors was, on its own, associated with adverse outcomes in trauma survivors. Analysis with LDlink showed positive LD for the three SNPs on chromosome 8 (rs2241777 vs. rs3098223: *D*′ = 0.99, *R* ^2^ = 0.82, *p* < 0.0001; rs2241777 vs. rs3134287: *D*′ = 0.99, *R* ^2^ = 0.81, *p* < 0.0001; rs3098223 vs. rs3134287: *D*′ = 0.99, *R* ^2^ = 0.97, *p* < 0.0001; **Supplementary Figure S1A**). Analysis of the three SNPs on chromosome 11 showed no LD (rs2065418 vs. rs10790334: *D*′ = 0.05, *R* ^2^ = 0.002, *p* = 0.005; rs2065418 vs. rs10741668: *D*′ = 0.03, *R* ^2^ = 0.0001, *p* = 0.4; rs10790334 vs. rs10741668: *D*′ = 0.009, *R* ^2^ = 0, *p* = 0.9; **Supplementary Figure S1B**). We also calculated *R* ^2^ manually for our population of blunt trauma patients, showing an even stronger positive correlation for the SNPs on chromosome 8, confirming the results of LDlink (rs2241777 vs. rs3098223: *R* ^2^ = 0.94; rs2241777 vs. rs3134287: *R* ^2^ = 0.01; rs3098223 vs. rs3134287: *R* ^2^ = 0.97). The SNPs on chromosome 11 showed similar low correlation coefficients as in the analyses with LDlink (rs2065418 vs. rs10790334: *R* ^2^ = 0.94; rs2065418 vs. rs10741668: *R* ^2^ = 0.007; rs10790334 vs. rs10741668: *R* ^2^ = 0.001), and therefore did not exhibit any LD among each other. Furthermore, this analysis showed that our cohort of blunt trauma patients is representative of the broader population in terms of genetic profile.

### Blunt Trauma Survivors With a Broad ISS Range Carrying Solely the rs2065418 TT Genotype Exhibit Trends Towards Worse Clinical Outcomes

According to our results above, we created single-SNP groups for the four non-linked SNPs (and excluding the other three SNPs) from our cohort of 413 blunt trauma survivors. This resulted in the following four groups: rs906790 TC only (*n* = 20 patients), rs2065418 TT only (*n* = 8 patients), rs10790334 TT only (*n* = 12 patients), and rs10741668 AA only (*n* = 22 patients). All four single SNP groups had comparable demographics (**Supplementary Table 2**), but only rs2065418 TT, located in the *MPPED2* gene, exhibited a trend towards worse outcomes (**Supplementary Figure S2**). We also compared the four groups with another group of patients carrying only the three linked SNPs in chromosome 8. There were no changes in our results: only rs2065418 TT showed trends to worse outcomes (data not shown).

### Blunt Trauma Survivors With a Broad ISS Range Carrying the rs2065418 TT Genotype Exhibit Significantly Higher MODScores Compared to Survivors Carrying the TG/GG Genotype

**Figure 1:**
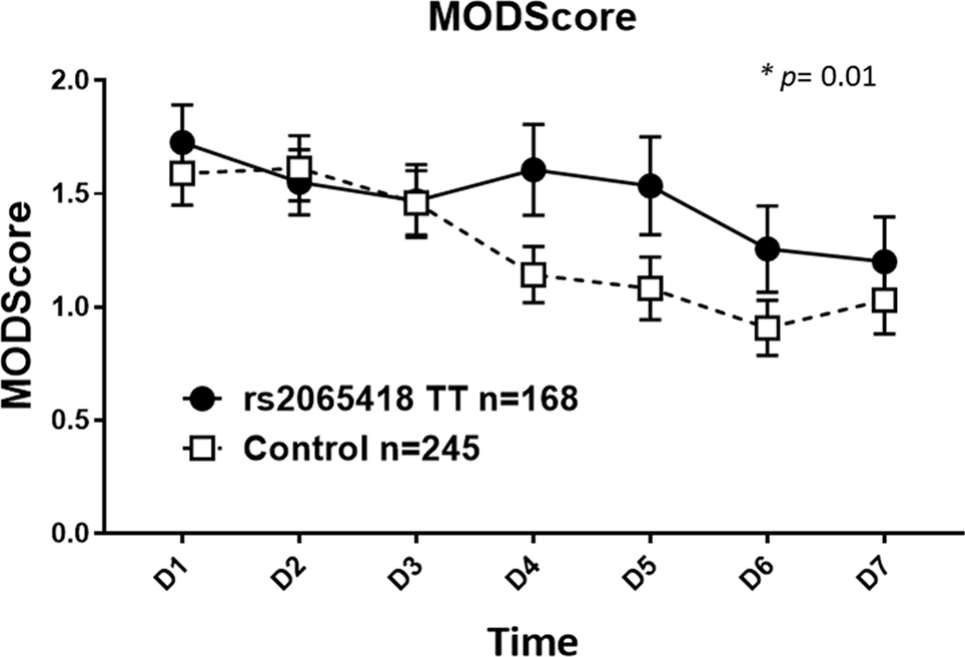
Significantly elevated Marshall MODScores in rs2065418 TT patients with a broad range of ISS vs. control. Rs2065418 TT patients with a broad range of ISS (*n* = 168) exhibited significantly higher Marshall MODScore values over 7 days (*p* = 0.01) compared to the control group of rs2065418 TG/GG patients (*n* = 245).

We next divided the initial blunt trauma cohort of 413 patients into two groups as follows: 1) rs2065418 TT, the genotype associated with non-survival (*n* = 168) and 2) rs2065418 TG/GG (*n* = 245). The demographics again were comparable (**Supplementary Table 3**). Rs2065418 TT blunt trauma survivors showed significantly elevated Marshall MODScores over a time course of the first week of hospitalization compared to the TG/GG control group (*p* = 0.01; **Figure 1**). The remaining assessed clinical outcomes [hospital length of stay (total LOS), ICU LOS, requirement for ventilation, and length of ventilation) showed trends towards worse outcomes in the rs2065418 TT group (**Supplementary Figure S3**).

### Severely Injured Blunt Trauma Survivors With the rs2065418 TT Genotype Have Significantly Worse Clinical Outcomes Compared to Survivors With a TG/GG Genotype

Given the putative roles of *MPPED2* in tissue homeostasis (Shen et al., 2016), as well as its association with mechanical ventilator-induced lung injury (Kompass et al., 2010), we next hypothesized that a certain injury severity threshold must be exceeded in order to observe a greater impact of rs2065418 TT on blunt trauma outcomes.

Accordingly, we stratified the 94 severely injured (commonly defined as ISS ≥ 25; Almahmoud et al., 2015) blunt trauma patients of the original population based on their rs2065418 genotype. The resultant two groups were as follows: 1) rs2065418 TT group, the genotype associated with non-survival [*n* = 42; age: 43.1 ± 3.1 years (min: 18 years, max: 89 years); ISS: 35.6 ± 1.3 (min: 25, max: 54); gender: 14 females, 28 males; ethnicity: 36 Caucasians, 3 African-Americans, 1 Asian, 2 unknown] and 2) rs2065418 TG/GG control group [*n* = 52; age: 41.6 ± 2.6 years (min: 18 years, max: 82 years), *p* = 0.71; ISS: 33.3 ± 0.9 (min: 25, max: 47), *p* = 0.27; gender: 12 females, 40 males, *p* = 0.35; ethnicity: 51 Caucasians, 0 African-American, 1 Asian, *p* = 0.13] (**Supplementary Table 4**). Furthermore, the groups were similar in AIS distribution and preexisting comorbidities (**Supplementary Figures S4** and **S5**). We confirmed that these patients carried the correct rs2065418 genotype using real-time PCR (data not shown).

**Figure 2:**
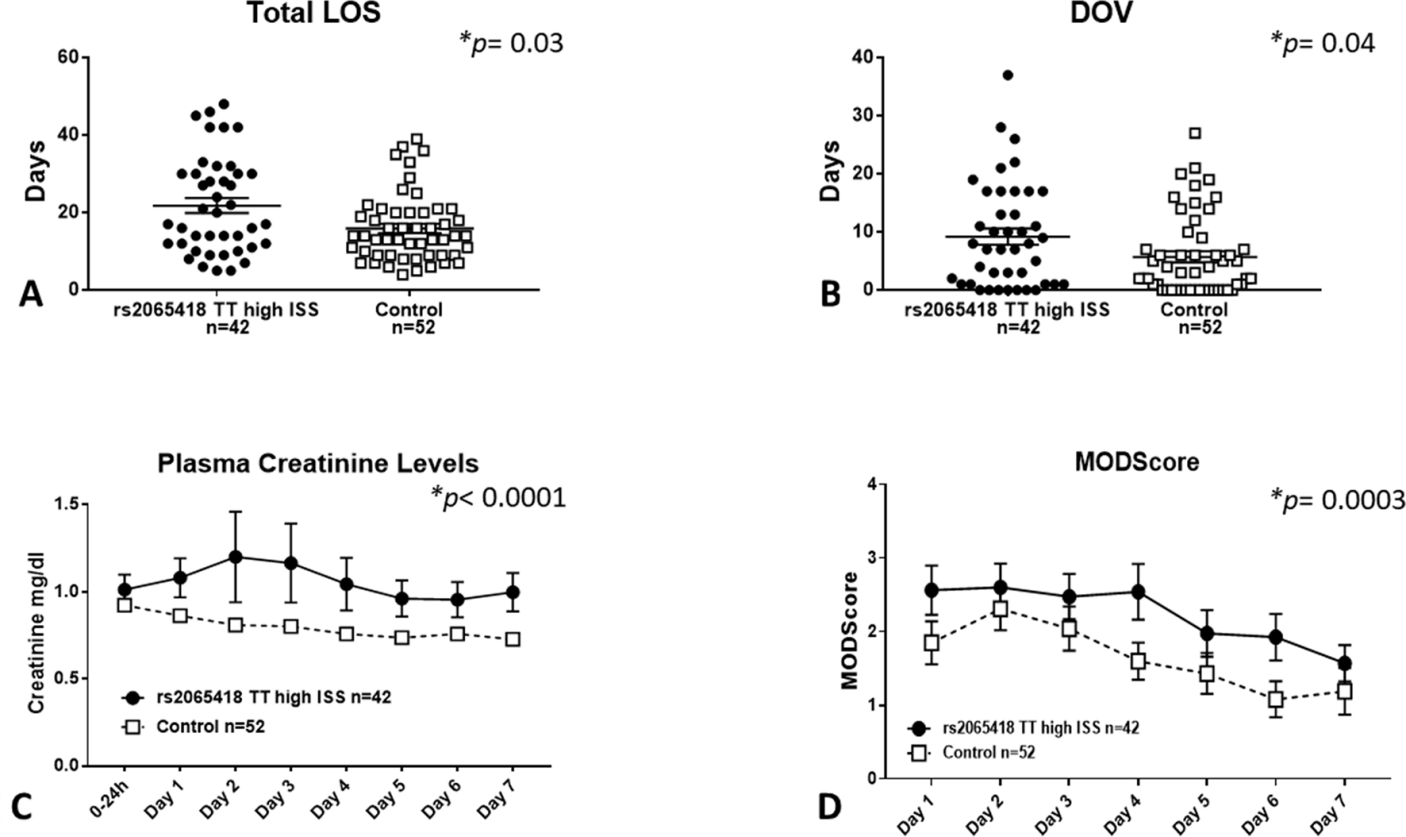
Severely injured rs2065418 TT patients exhibit worse clinical outcomes vs. control. High-severity rs2065418 TT patients (*n* = 42) and control rs2065418 TG/GG patients (*n* = 52) were assessed for clinical outcomes as described in the *Materials and Methods*. High-ISS rs2065418 TT patients exhibited longer total LOS (*p* = 0.03) **(A)** longer DOV (*p* = 0.04) **(B)** elevated plasma creatinine levels (*p* 0.0001) **(C)**, and higher Marshall MODScore over 7 days (*p* = 0.0003) **(D)** compared to the control group of high-ISS rs2065418 TG/GG patients. Total LOS and DOV were not normally distributed and therefore were tested by Mann–Whitney *U* test.

Severely injured patients carrying the rs2065418 TT genotype exhibited significantly longer total LOS (21.7 ± 1.9 days) vs. the control group of rs2065418 TG/GG patients (LOS: 15.9 ± 1.2 days, *p* = 0.03) (**Figure 2A**). Furthermore, high-ISS rs2065418 TT patients were on mechanical ventilation significantly longer (DOV: 9.2 ± 1.4 days vs. 5.7 ± 1 days in controls; *p* = 0.04) and had higher plasma creatinine levels over the first 7 days compared to the control group (*p* < 0.0001) (**Figures 2B, C**). The rs2065418 TT patients also had higher Marshall MODScores over the first 7 days (*p* = 0.0003) (**Figure 2D**). The requirement for ventilation was nearly statistically significant (*p* = 0.15), as were the rates of nosocomial infections [57% (rs2065418 TT) vs. 40% (Control); *p* = 0.14]. Analysis of patients carrying the TG genotype of rs2065418 vs. patients with the GG genotype did not show any significant differences in these same parameters (data not shown).

### Severely Injured rs2065418 TT Patients Exhibit Distinct Inflammatory Responses From Those of rs2065418 TG/GG Control Patients

In our prior study, trauma survivors carrying all seven non-survivor SNPs exhibited different inflammatory responses from survivors having none of those SNPs (Schimunek et al., 2018). Accordingly, we next compared the dynamic changes in the circulating levels of 31 inflammatory mediators in the first 7 days after injury between high-ISS rs2065418 TT patients and control (high-ISS TG/GG). Nine of the 31 assessed circulating inflammatory mediators showed statistically significant (*p* ≤ 0.01) differences between rs2065418 TT patients and rs2065418 TG/GG controls: rs2065418 TT patients expressed higher levels of Eotaxin (*p* = 0.003) and MCP-1 (*p* = 0.0003) vs. rs2065418 TG/GG control patients and lower plasma levels of IFN-α (*p* = 0.007), IL-2 (*p* = 0.008), IL-4 (*p* = 0.001), IL-9 (*p* < 0.0001), IL-15 (*p* = 0.008), IL-17A (*p* < 0.0001), and IL-23 (*p* = 0.007) (**Table 1** and **Supplementary Figure S6**).

**Table 1:** Significantly different inflammatory mediators in severely injured rs2065418 TT patients (*n* = 42) vs. control (*n* = 52).

	Inflammatory mediators	*p* value	Rs2065418 TT high ISS Mean ± SEM (pg/ml)	Control high ISS Mean ± SEM (pg/ml)
**Elevated in rs2065418 TT high ISS**	Eotaxin	0.003	55.3 ± 2.4	46.2 ± 1.4
	MCP-1	0.0003	985.7 ± 72.1	659.4 ± 40.2
**Lowered in rs2065418 TT high ISS**	IFN-α	0.007	67.3 ± 4.2	85.9 ± 5.1
	IL-2	0.008	12 ± 1.5	17.8 ± 1.9
	IL-4	0.001	52.6 ± 3.8	71 ± 4.3
	IL-9	<0.0001	4.8 ± 0.6	10.9 ± 1.0
	IL-15	0.008	36.1 ± 3.6	52.7 ± 5.7
	IL-17A	<0.0001	61 ± 5.7	96 ± 6.7
	IL-23	0.007	1103 ± 124.1	1712 ± 140.3

Plasma levels of 31 inflammatory mediators were assessed as described in the Materials and Methods, showing that high-ISS rs2065418 TT patients (n = 42) exhibited two significantly elevated mediators over a time of 7 days (Eotaxin and MCP-1) vs. control (n = 52), while seven inflammatory mediators were significantly lower (IFN-α, IL-2, IL-4, IL-9, IL-15, IL-17A, and IL-23).

### Severely Injured rs2065418 TT Patients Exhibit Differential Inflammatory Mediator Connectivity and Network Complexity Over 7 Days Following Injury as Compared to rs2065418 TG/GG Control Patients

Rising network complexity is associated with elevated MODScores in the first 5 days after trauma (Namas et al., 2016a), which is in turn associated with the set of seven SNPs that include rs2065418 TT (Schimunek et al., 2018). Accordingly, we hypothesized that severely injured patients that carry the rs2065418 TT genotype and exhibit higher MODScores in the first 7 days (**Figure 2D**) would exhibit a higher NCS than control.

**Figure 3:**
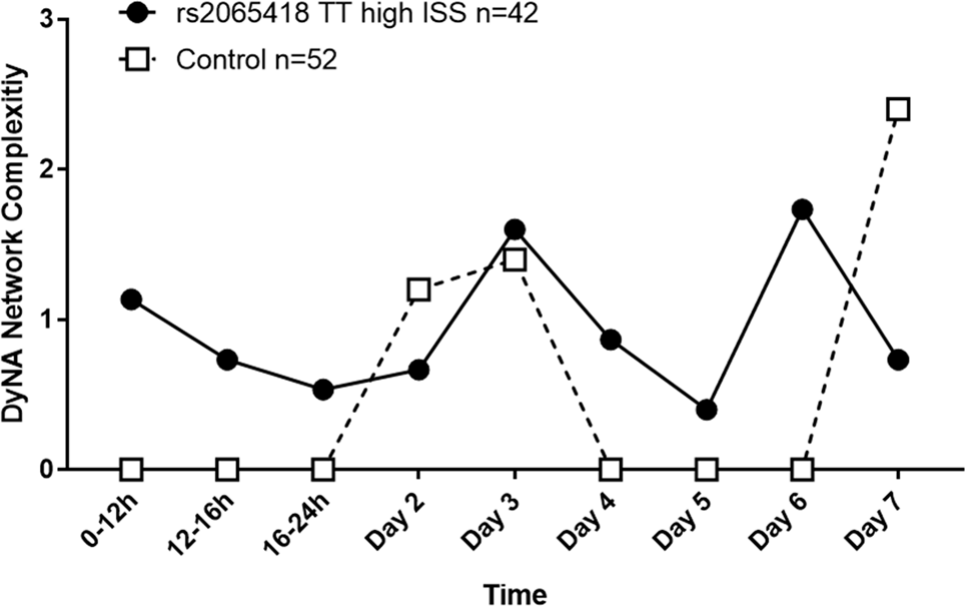
Analysis of network complexity shows an initially elevated inflammatory response in severely injured rs2065418 TT patients. DyNA of inflammatory mediators was carried out using data from admission to 7 days following injury, as described in the *Materials and Methods*. This analysis revealed that severely injured rs2065418 TT patients (*n* = 42) exhibited elevated inflammatory network complexity score (NCS) values in the first 24 h relative to controls (*n* = 52). rs2065418 TT and control TG/GG patients reached comparable NCS values at days 2 and 3. During days 4 to 6, rs2065418 TT patients again showed higher NCS values vs. controls. At day 7, control patients exhibited an elevated NCS compared to rs2065418 TT patients.

Indeed, high-ISS rs2065418 TT patients exhibited more complex inflammatory networks in the first 24 h following hospital admission relative to controls (NCS: 0.5–1.2 vs. 0), though both TT and control TG/GG patients reached similar levels by day 3 (NCS: 1.6 vs. 1.4). During days 4 to 6, high-ISS rs2065418 TT patients again showed higher NCS (0.4–1.7 vs. 0). At day 7, control patients exhibited an elevated complexity score compared to rs2065418 TT patients (NCS: 0.7 vs. 2.4) (**Figure 3** and **Supplementary Table 5**).

We next examined the network connectivity of each inflammatory mediator assessed in order to help define dominant pathways associated with each rs2065418 genotype. The total number of connections observed in rs2065418 TG/TT control patients was 45% lower compared to high-ISS rs2065418 TT patients (260 vs. 144 total connections) over a time course of 7 days after admission (**Supplementary Table 5**). In the rs2065418 TT group, the most connected mediators (defined as the third quartile of total connections in their respective patient subgroups) were GM-CSF, IL-2, IL-1β, IFN-α, IL-4, MIP-1β, IL-17A, and sIL-2Rα (third quartile: 16). In contrast, IL-2, IL-1β, IFN-α, IFN-γ, IL-15, GM-CSF, IL-17A, and IL-4 were the most connected mediators in the control group (third quartile: 9) (**Supplementary Figure S7** and **Supplementary Table 5**).

### No Clinical Outcome Differences in Severely Injured Blunt Trauma Survivors Stratified by a Control Single-Nucleotide Polymorphism

Given the restricted number of patients in our population and the possibility that the differences we observed based on rs2065418 genotype might be due to random chance, we applied the same principle of dividing severely injured patients based on a random SNP. The SNP we used was rs7705676 (Chr 5; p7; pos 35,237,634; base T/C; MAF C = 0.31), which is not associated with any gene and for which there are no publications based on a PubMed search at the time of this report.

As before, we stratified the 94 severely injured (ISS ≥ 25) blunt trauma patients based on their genotype. The resultant two groups were as follows: 1) rs7705676 TT group [*n* = 27; age: 44.9 ± 3.8 years (min: 18 years, max: 83 years); ISS: 33.6 ± 1.5 (min: 25, max: 50); gender: 6 females, 21 males; ethnicity: 25 Caucasians, 2 African-Americans, 0 Asians] and 2) rs7705676 TC/CC control group [*n* = 67; age: 41.2 ± 2.3 years (min: 18 years, max: 89 years), *p* = 0.38; ISS: 34.6 ± 0.9 (min: 25, max: 54), *p* = 0.53; gender: 20 females, 47 males, *p* = 0.61; ethnicity: 52 Caucasians, 1 African-American, 2 Asians, 2 unknown]. Additionally, the groups were similar in AIS distribution and preexisting comorbidities.

We first compared clinical outcomes (total LOS, ICU LOS, DOV, requirement for ventilation, and Marshall MODScore) between rs7705676 TT high ISS and the rs7706576 TC/CC control group, which yielded no differences (data not shown). Furthermore, as with rs2065418, we compared the dynamic changes in the circulating levels of 31 inflammatory mediators in the first 7 days after injury between high-ISS rs7705676 TT patients and control (high-ISS TC/CC). In high-ISS rs7705676 TT patients, IL-9 (*p* = 0.0005) and IL-21 (*p* = 0.006) were significantly lower vs. high-ISS rs7705676 TC/CC control patients (data not shown).

## Discussion

The present study investigated a SNP in the *MPPED2* gene associated with altered inflammation and adverse clinical outcomes in severe blunt trauma. Severely injured patients (ISS ≥ 25) carrying the TT genotype of the *MPPED2* SNP rs2065418 exhibited greater hospital LOS, higher MODScores over time, longer time on mechanical ventilation, elevated plasma levels of creatinine over time, and alterations of systemic inflammation.

Over the past 30 years, intense research on the immunological response of trauma has led to a growing list of immune cell populations and inflammatory mediators linked with adverse outcomes (Lord et al., 2014; Namas et al., 2015). More recently, the field of trauma research has been extended into genomics and the potential role of individual genetic variability, in the form of SNPs, on trauma outcomes (Hildebrand et al., 2011; Bronkhorst et al., 2015). As yet, however, there is a lack of unbiased studies on SNPs associated trauma outcomes.

In prior work, we introduced a new enrichment strategy that allowed us to carry out a relatively unbiased screen for SNPs in a large cohort of trauma patients. This effort identified a set of seven SNPs associated with an altered inflammatory response and non-survival post-trauma (Schimunek et al., 2018). Herein, we demonstrate that one of those SNPs, rs2065418 (Chr 11; p14.1; Pos 30,400,521; T/G), which is located in an intron region of the *MPPED2* gene, appears to stratify the clinical outcomes and inflammatory trajectories of blunt trauma patients, especially when severely injured. Due to its location in the gene, the SNP could affect the level of protein expression or change the function of the protein by altering the splicing process (Syvanen, 2001).

The MPPED2 protein has phosphodiesterase activity and can degrade cAMP and cGMP, inhibiting cyclic nucleotide signaling. Additionally, *MPPED2* might act as scaffolding or adaptor protein (Tyagi et al., 2009; Dermol et al., 2011). Inhibiting *MPPED2* resulted in increased proliferation and migration of oral squamous cell carcinoma, suggesting anti-proliferative functions of the *MPPED2* gene product (Shen et al., 2016). On the other hand, an increased expression of *MPPED2* in tumor cell lines arrested the cell cycle of those cells and therefore impaired the proliferation. Furthermore, an upregulated expression of *MPPED2* was associated with better outcomes in neuroblastoma patients (Liguori et al., 2012).

A previous genome-wide association study suggested that *MPPED2* influences the estimated glomerular filtration rate (eGFR) and appears to be associated with renal function in patients with chronic kidney disease (Pattaro et al., 2012). Another study associated *MPPED2* with the pathobiology of experimental ventilator-induced lung injury (Kompass et al., 2010). We therefore speculate that SNPs in the *MPPED2* gene, such as the TT genotype of rs2065418 based on our previous (Schimunek et al., 2018) and present work, possibly impacts both renal and lung function of trauma patients. Because of its putative anti-proliferative function, it is also possible that the rs2065418 TT genotype impairs tissue repair in trauma patients. However, the molecular explanations for the observed association of the rs2065418 SNP remain unknown.

**Figure 4:**
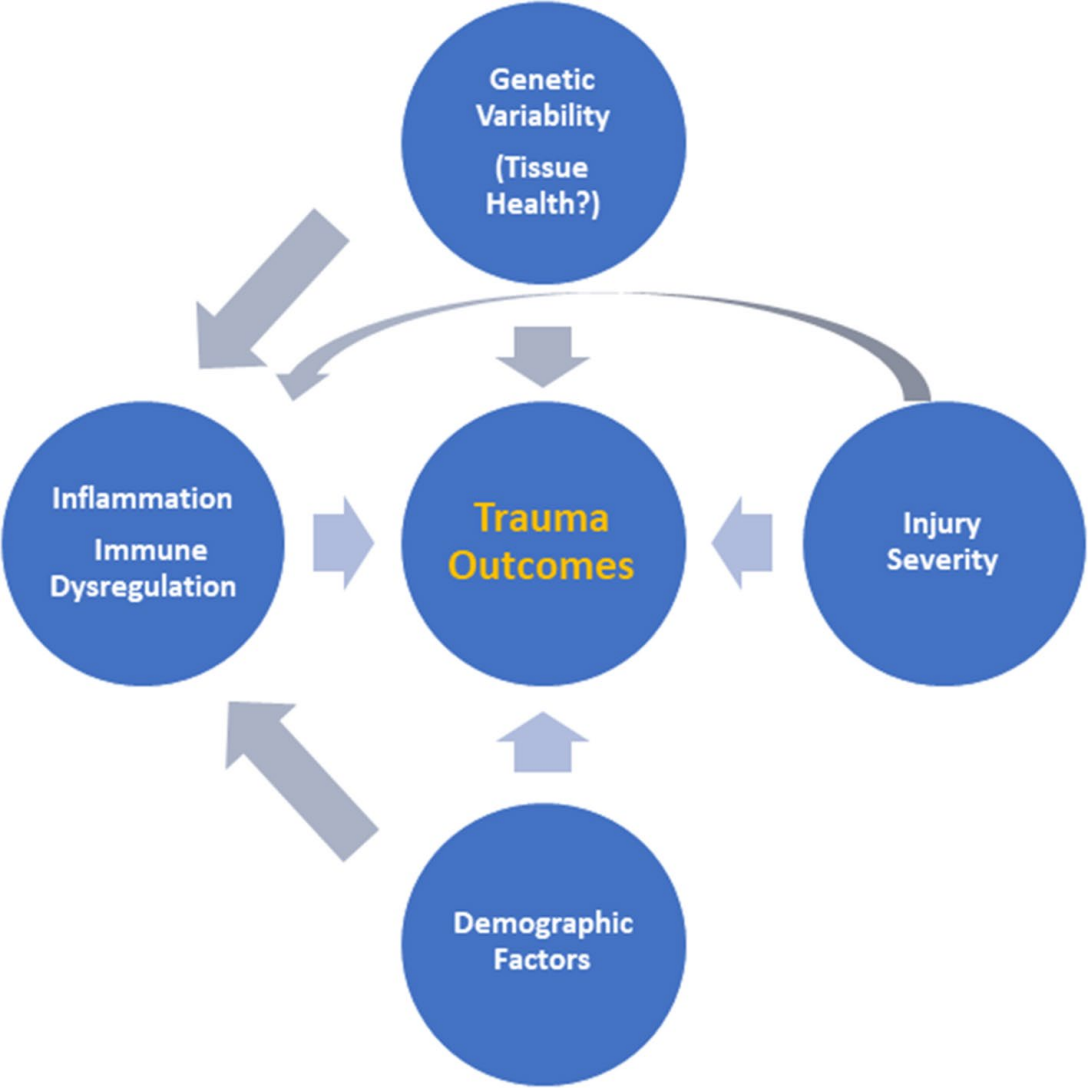
Putative interactions among injury severity, inherent tissue resilience, and the systemic inflammatory response. Based on the results presented in this study, we suggest an intertwined interaction among injury severity, inherent (genetically determined) tissue resilience, and the systemic inflammatory response driving outcomes after trauma as a novel paradigm for understanding the response to traumatic injury.

Taken together, these previously published studies suggest that *MPPED2* is likely related to tissue health and metabolism rather than inflammation. This could explain why we could only find an association between rs2065418 genotype and systemic inflammation in the most severely injured patients. It seems possible that the effects of rs2065418 TT on a moderately injured population of trauma patients (ISS ∼ 20) are too minimal to directly affect outcomes such as duration of mechanical ventilation, although this genotype is associated with multiple organ dysfunction during hospitalization. Our studies thus suggest an intertwined interaction among injury severity, inherent (genetically determined) tissue resilience, and the systemic inflammatory response to these combined stimuli (**Figure 4**), which may form a novel paradigm for gene–environment interactions in the setting of traumatic injury.

Differential *MPPED2* genotype may also impact trauma outcomes due to lung–kidney crosstalk, a well-known phenomenon in critical illness, with acute lung injury affecting kidney function and *vice versa* (Domenech et al., 2017). It is unclear at this point if the impaired kidney function and extended need for respiratory support observed in high-ISS rs2065418 TT patients are two independent phenomena or if one causes the other. We speculate that *MPPED2* is a potential player in lung–kidney crosstalk by worsening lung and/or kidney injury and/or altering the inflammatory response.

In addition to the differences in clinical outcomes, we observed altered plasma levels of 11 inflammatory mediators in high-ISS rs2065418 TT patients vs. controls, of which eight were decreased. Furthermore, the third quartile of total connections for individual mediators in the network analysis was higher in high-ISS rs2065418 TT patients compared to control. Blunt trauma patients who go on to develop nosocomial infections (Namas et al., 2016b), as well as patients who present with an elevated base deficit indicative of metabolic dysfunction following trauma (Abdul-Malak et al., 2016), also exhibit a high, sustained network connectivity. Thus, to date, elevated dynamic inflammatory network complexity has been associated with worse clinical phenotypes. According to these prior observations, severely injured rs2065418 TT patients exhibited higher initial inflammatory network complexity. How the rs2065418 SNP in the *MPPED2* gene selectively regulates components of the immune and inflammatory responses is unclear, but may be related to the extent of tissue injury and repair.

There are several limitations of the study to consider. One major limitation is the size of the patient subgroups. Clearly, our findings must be confirmed in a larger and more diverse trauma patient population, ideally in a multicenter study. Another limitation is the number of inflammatory parameters assessed in our analysis.

In conclusion, we suggest that the identification of the rs2065418 TT genotype in the *MPPED2* gene with adverse outcomes in the most severely injured trauma patients could be useful to screen for populations at risk of injury or in patients following severe injury. Additional studies will be required to determine the potential of this SNP as a stratification biomarker.

## Ethics Statement

This study was carried out in accordance with the recommendations of the Institutional Review Board (IRB) of the University of Pittsburgh with written informed consent from all subjects. All subjects gave written informed consent in accordance with the Declaration of Helsinki. The protocol was approved by the Institutional Review Board (IRB) of the University of Pittsburgh.

## Author Contributions

LS and RZ participated in data analysis and manuscript writing. JY, DB, and FE-D performed experiments. RN, DL, AA, and MC participated in data analysis. TB obtained funding and participated in manuscript writing. YV obtained funding, participated in data analysis, manuscript writing, and coordinated the overall research effort.

## Funding

This work was supported by Department of Defense contracts W81XWH-15-PRORP-OCRCA and W81XWH-1820051. This research was supported in part by a grant from the National Institutes of Health (T32GM075770).

## Conflict of Interest

The contents of this manuscript are included in a patent application (U.S. Patent Application No. 15/971,519), on which LS, RN, TB, and YV are listed as co-inventors and which has been assigned to the University of Pittsburgh. 

The remaining authors declare that the research was conducted in the absence of any commercial or financial relationships that could be construed as a potential conflict of interest.

## Abbreviations

ANOVA, analysis of variance; ATP, adenosine triphosphate; DCAF, DDB1- and CUL4-associated factor; eGFR, estimated glomerular filtration rate; FAD, Flavin adenine dinucleotide; GM-CSF, granulocyte-macrophage colony-stimulating factor; IFN, interferon; IL, interleukin; IL-1RA, IL-1 receptor antagonist; IP-10, interferon-γ-inducible protein of 10 kDa; LD, linkage disequilibrium; MIG, monokine induced by interferon-γ; MIP, macrophage inflammatory protein; MPPED2, metallophosphoesterase domain containing protein 2; NCS, Network Complexity Score; sIL-2Rα, soluble interleukin-2 receptor α-chain; SLC25A32, solute carrier family 25 member 32; sST2, Soluble ST2, IL-1 receptor-like 1; Th17, T helper 17 cells; TNF-α, tumor necrosis factor-α.
