# Supplementary material for: MPPED2 Polymorphism Is Associated With Altered Systemic Inflammation and Adverse Trauma Outcomes
Source: Front Genet. 2019 Nov 8;10:1115. doi: 10.3389/fgene.2019.01115 (PMC6857553; doi:10.3389/fgene.2019.01115)
Supplement: Supplementary file 1 [file DataSheet_1.docx]

**SUPPLEMENTAL METHODS**

**DNA analysis using Illumina® arrays**

Whole blood samples were collected into heparinized tubes. DNA was extracted using the QIAamp® DNA Blood Midi Kit (QIAGEN, Valencia, CA) as per manufacturer’s specifications. Single nucleotide polymorphism genotyping was performed with 200 ng of genomic DNA input using the Human Core Exome-24 v1.1 BeadChip (Illumina, San Diego, CA) following the manufacturer’s Infinium® HTS Assay protocol. Briefly, DNA was denatured in 0.1N NaOH and neutralized prior to isothermal amplification. Amplified DNA was fragmented and then hybridized to locus-specific 50mers that make up the array for 16-24 h with rocking at 48˚C. After removal of unbound or non-specifically annealed DNA, single base extension of the 50mer oligonucleotides was performed with labeled nucleotides, which were scanned using an Illumina iScan with autoloader 2.x. Data analysis was performed using Illumina Genome Studio 2.0.

**Real-time PCR**

Real-time PCR was carried out in 96-well FG-Microplates using Applied Biosystems kits (TaqMan SNP genotyping assay and TaqPath ProAmp master mixes, Waltham, MA). The reactions were carried out using 20 ng of human genomic DNA in a total volume of 20 µl. PCR involved standard cycles including an initial 95 °C for 12 min and 40 cycles of denaturation at 95 °C for 15 sec, and annealing/extension at 60 °C for 1 min using a 7900HT Fast Real-Time PCR system (Applied Biosystems, Waltham, MA). Endpoint reads were done on the plates. Results were analyzed using the Applied Biosystems Software version SDS2.4.

**Serial analysis of inflammatory mediators**

Whole blood samples were withdrawn in heparinized tubes 3 times in the first 24 h after admission, and then daily for 7 days. The samples were kept on ice and centrifuged to obtain plasma, and then stored at -80 °C until assayed for inflammatory mediators. The Luminex™ 100 IS analyzer (Luminex, Austin, TX) and Human Cytokine/Chemokine MILLIPLEX™ Panel kit (Millipore Corporation, Billerica, MA) were used to measure plasma levels of Eotaxin (CCL11), interleukin (IL)-1β, IL-1 receptor antagonist (IL-1RA), IL-2, soluble IL-2 receptor-α (sIL-2Rα), IL-4, IL-5, IL-6, IL-7, IL-8 (CCL8), IL-10, IL-13, IL-15, IL-17A, interferon (IFN)-α, IFN-γ, IFN-γ inducible protein (IP)-10 (CXCL10), monokine induced by gamma interferon (MIG; CXCL9), macrophage inflammatory protein (MIP)-1α (CCL3), MIP-1β (CCL4), monocyte chemotactic protein (MCP)-1 (CCL2), granulocyte-macrophage colony stimulating factor (GM-CSF), and tumor necrosis factor alpha (TNF-α). Human Th17 MILLIPLEX™ Panel kit (Millipore Corporation, Billerica, MA) was used to measure IL-9, IL-21, IL-22, IL-23, IL-17E/25, and IL-33. Nitrite/Nitrate (NO_2_^-^/NO_3_^-^) levels were measured by a Griess Reagent colorimetric assay (Cayman Chemical, Ann Arbor, MI). Plasma levels of soluble ST2 (sST2) were measured by a sandwich ELISA assay (R&D Systems, Minneapolis, MN). All cytokine/chemokine mediator concentrations are given in pg/ml; NO_2_^-^/NO_3_^-^ concentrations are in µM. Experimental data are shown as mean ± SEM.

**Dynamic Network Analysis**

Dynamic Network Analysis (DyNA) (Mi et al., 2011) was used to define the central

inflammatory network mediators as a function of both time and patient sub-group. Using inflammatory mediator measurements of at least three time-points for experimental group, networks were created at nine consecutive time periods (0-12h, 12-16h, 16-24h, Day 2, Day 3, Day 4, Day 5, Day 6, and Day 7) using Matlab® software. Connections ([network edges] represent trajectories of inflammatory mediators [network nodes] that move in parallel; positive: same direction; negative: opposite direction) were created if the Pearson correlation coefficient between any two nodes (inflammatory mediators) at the same time-interval was greater or equal to a threshold of 0.85, as indicated. The network complexity for each time-interval was calculated using the following formula: Sum (N1 + N2 +…+ N_n_)/n-1, where N represents the number of connections for each mediator and n is the total number of mediators analyzed. The total number of connections represents the sum of the number of connections across all time intervals for all patients in a given sub-group. In previous studies, we showed, that rising network complexity is associated with rising MODScores when comparing trauma survivors vs non-survivors (Abboud et al., 2016).

**SUPPLEMENTAL FIGURE LEGENDS**

**Supplemental Figure 1: Testing for linkage disequilibrium shows strong correlations between** **rs2241777, rs3098223 and rs3134287, all located on chromosome 8**

The SNPs located on chromosome 8 (rs2241777, rs3098223 and rs3134287) showed strong linkages between each other (D’= 0.99, *p*-value< 0.0001 for all comparisons) (A), while the three SNPs on chromosome 11 (rs2065418, rs10741668 and rs10790334) showed no linkage (B). Additionally, the spatial relationship is depicted for the SNPs on chromosome 8, for the SNPs on chromosome 11 the distance was too big to plot them (*“too many genes to plot”*).

**Supplemental Figure 2: Analysis of individual non-survival-associated SNPs groups points to rs2065414 TT**

Comparison of clinical outcomes (total LOS [A], ICU LOS [B], DOV [C], requirement for mechanical ventilation [D] and MODScore [E]) across individual non-survival SNPs (rs906790 TC [n=20], rs2065418 TT [n=8], rs10790334 TT [n=12] and rs10741668 AA [n=22]. Though not statistically significant, there were clear trends towards statistically significant differences in patients with rs2065414 TT. Data in A-D were not normally distributed and therefore tested by Kruskal-Wallis test, followed by Dunn's multiple comparisons test.

**Supplemental Figure 3: No significantly different clinical outcomes between rs2065418 TT patients with a broad range of ISS vs. controls**

rs2065418 TT patients with a broad range of ISS (n=138) exhibited no statistically different clinical outcomes (total LOS [A], ICU [B], DOV [C], requirement for ventilation [D], and MODScore [E]) vs control (n=246). The data in A-D were not normally distributed and therefore tested by Kruskal-Wallis test, followed by Dunn's multiple comparisons test.

**Supplemental Figure 4: Comparable scores of the Abbreviated Injury Scale between severely injured rs2065418 TT patients (n= 35) vs. controls (n= 49)**

No statistical significance was observed across the six body regions that comprise the ISS (head, face, chest, abdomen, extremities/pelvis, and external), measured via Abbreviated Injury Scale (AIS). The data were not normally distributed for all six body regions and therefore tested by Mann-Whitney U test.

**Supplemental Figure 5: Comparable comorbidities between severely injured rs2065418 TT patients (n= 35) vs. controls (n= 49)**

Abbreviations: Hypertension (HTN), Diabetes mellitus (DM), Coronary artery disease (CAD), Myocardial infarction (MI), coronary artery bypass graft (CABG), Chronic obstructive pulmonary disease (COPD), Cardiovascular disease (CVD), Pulmonary embolism (PE), Deep vein thrombosis (DVT), Gastroesophageal reflux disease (GERD), Inflammatory bowel disease (IBD), Hepatitis C Virus (HCV).

**Supplemental Figure 6: Significantly different inflammatory mediators of severely injured rs2065418 TT patients vs. controls**

High ISS rs2065418 AA patients (n= 35) exhibited 3 significantly elevated mediators over a time course of 7 days (Eotaxin, MCP-1 and MIP) vs. control (n= 49) while 7 inflammatory mediators were significantly decreased (GM-CSF, IFN-α, IL-4, IL-9, IL-15, IL-17A, and IL-23).

**Supplemental Figure 7: Heat map showing the network connectivity of individual inflammatory mediators reveals impaired connectivity in high severity rs2065418 TT patients over the first 7 days following injury**

DyNA was carried out, followed by plotting the resulting connectivity for each inflammatory mediator in heat maps for high ISS rs2065418 TT patients (n= 35) and control rs2065418 TG/GG patients (n= 49), as described in the *Materials and Methods*. Color bar indicates number of connections per inflammatory mediator. The heat maps visualize the impaired connectivity in high ISS rs2065418 AA patients over the first 7 days following injury. The inflammatory mediators are ranked by total connectivity over time, and the most connected mediators (defined as the upper 3^rd^ quartile of total connections in their respective patient sub-groups) are highlighted in red boxes. In high ISS rs2065418 TT patients, the most connected mediators were GM-CSF, IL-1β, IFN-α, IL-2, IL-33, MIP-1β, IL-23, and IL-17A (3^rd^ quartile: 17.5). In contrast, IL-2, IL-1β, IL-15, IL-17A, MIP-1β, MIP-1α, IFN-α, IL-33, and IFN-γ were the most connected mediators in the control group (3^rd^ quartile: 25).

**SUPPLEMENTAL TABLE LEGENDS**

**Supplemental Table 1: Individual SNPs Associated with Trauma Non-survivors**

Overview over a set of seven SNPs associated with trauma non-survival (Schimunek et al., 2017) that are investigated further in this study.

**Supplemental Table 2: Demographics of single-SNP groups**

Analysis of demographics showed that age (years), injury severity score (ISS), and gender distribution were comparable across all groups. Age was distributed normally across the groups and therefor tested by One-Way ANOVA, followed by Tukey’s multiple comparison test. ISS was not normally distributed and therefore tested by Kruskal-Wallis test, followed by Dunn's multiple comparisons test.

**Supplemental Table 3: Demographics of** **rs2065418 TT patients with a broad range of ISS vs. controls**

Analysis of demographics between rs2065418 TT patients with a broad range of ISS vs. controls showed that Age (years) and Injury severity score (ISS) were comparable, the control group had a significantly higher ratio of male patients (*p* = 0.04). Age was distributed normally and therefore tested by Student’s t-test. ISS was not normally distributed and therefor tested by Mann-Whitney U test.

**Supplemental Table 4: Demographics of severely injured rs2065418 TT patients vs. control**

Comparison of demographics of high ISS rs2065418 patients vs. controls showed no statistical significance for age (years), injury severity score (ISS), and gender distribution. Age was distributed normally and therefor tested by Student’s t-test. ISS was not normally distributed and therefore tested by Mann-Whitney U test.

**Supplemental Table 5: Network connectivity for each mediator in high ISS rs2065418 TT patients vs. control**

The DyNA network connectivity for each mediator in both patient sub-groups is given in hierarchical order, ranked by total connections (A: Control [n= 49]; B: high ISS rs2065418 TT [n= 35]). The 3^rd^ quartile of total connectivity over time was 17.5 in high ISS rs2065418 TT patients vs. 25 in controls. Mediators exceeding that threshold are highlighted in the red boxes. The number of total connections over time was also lower in high ISS rs2065418 TT patients (346 vs. 480 in controls).

**REFERENCES**

Abboud, A., Namas, R.A., Ramadan, M., Mi, Q., Almahmoud, K., Abdul-Malak, O., et al. (2016). Computational Analysis Supports an Early, Type 17 Cell-Associated Divergence of Blunt Trauma Survival and Mortality. *Crit Care Med* 44(11)**,** e1074-e1081. doi: 10.1097/ccm.0000000000001951.

Mi, Q., Constantine, G., Ziraldo, C., Solovyev, A., Torres, A., Namas, R., et al. (2011). A dynamic view of trauma/hemorrhage-induced inflammation in mice: Principal drivers and networks. *PLoS ONE* 6**,** e19424.

Schimunek, L., Namas, R.A., Yin, J., Liu, D., Barclay, D., El-Dehaibi, F., et al. (2017). An Enrichment Strategy Yields Seven Novel Single Nucleotide Polymorphisms Associated with Mortality and Altered TH17 Responses Following Blunt Trauma. *Shock*. doi: 10.1097/shk.0000000000000987.
