# Supplementary material for: MPPED2 Polymorphism Is Associated With Altered Systemic Inflammation and Adverse Trauma Outcomes
Source: Front Genet. 2019 Nov 8;10:1115. doi: 10.3389/fgene.2019.01115 (PMC6857553; doi:10.3389/fgene.2019.01115)
Supplement: Supplementary file 2 [file Presentation_1.pptx]

## Slide 1
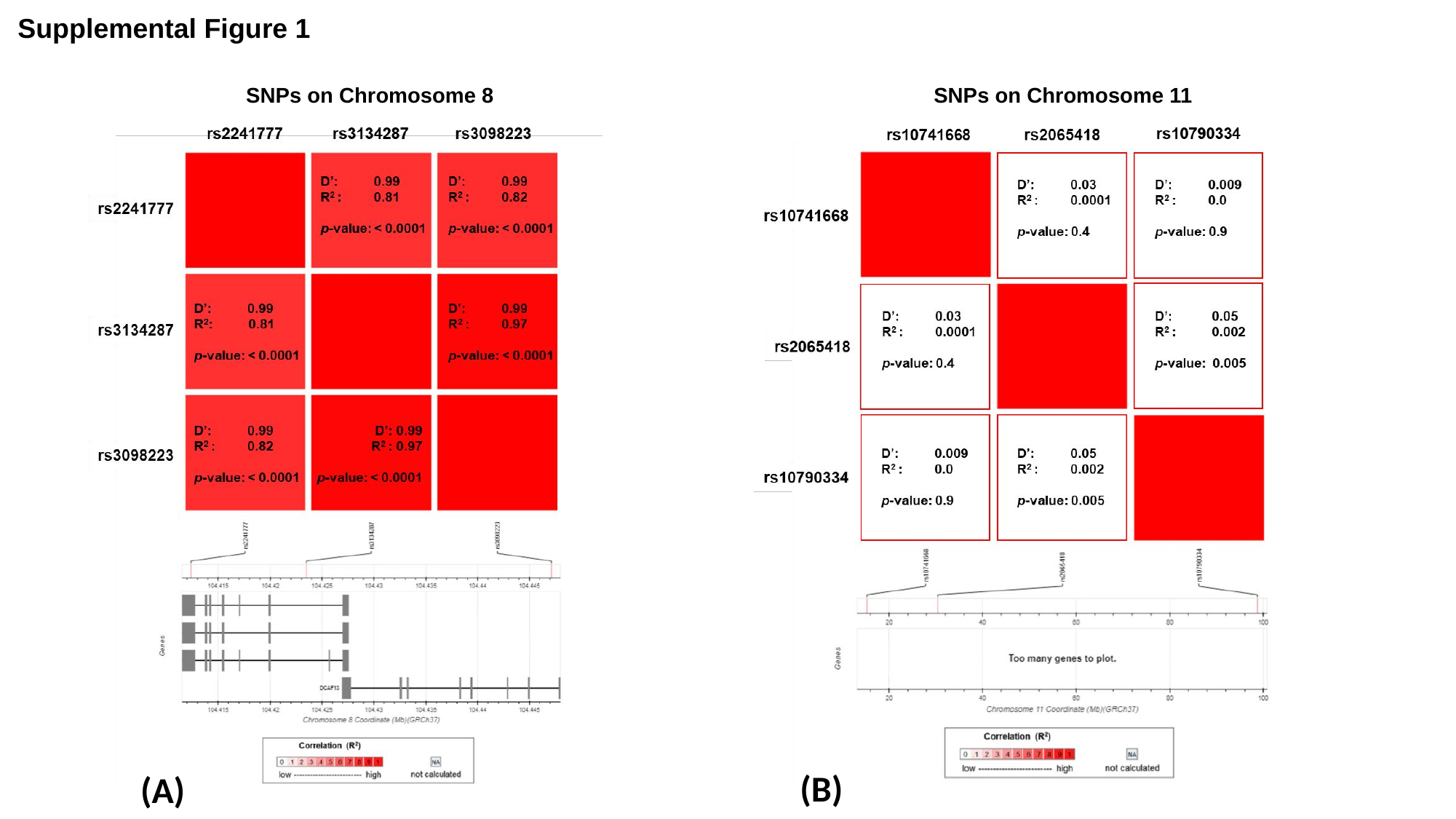

Supplemental Figure 1
SNPs on Chromosome 8
SNPs on Chromosome 11
(B)
(A)

## Slide 2
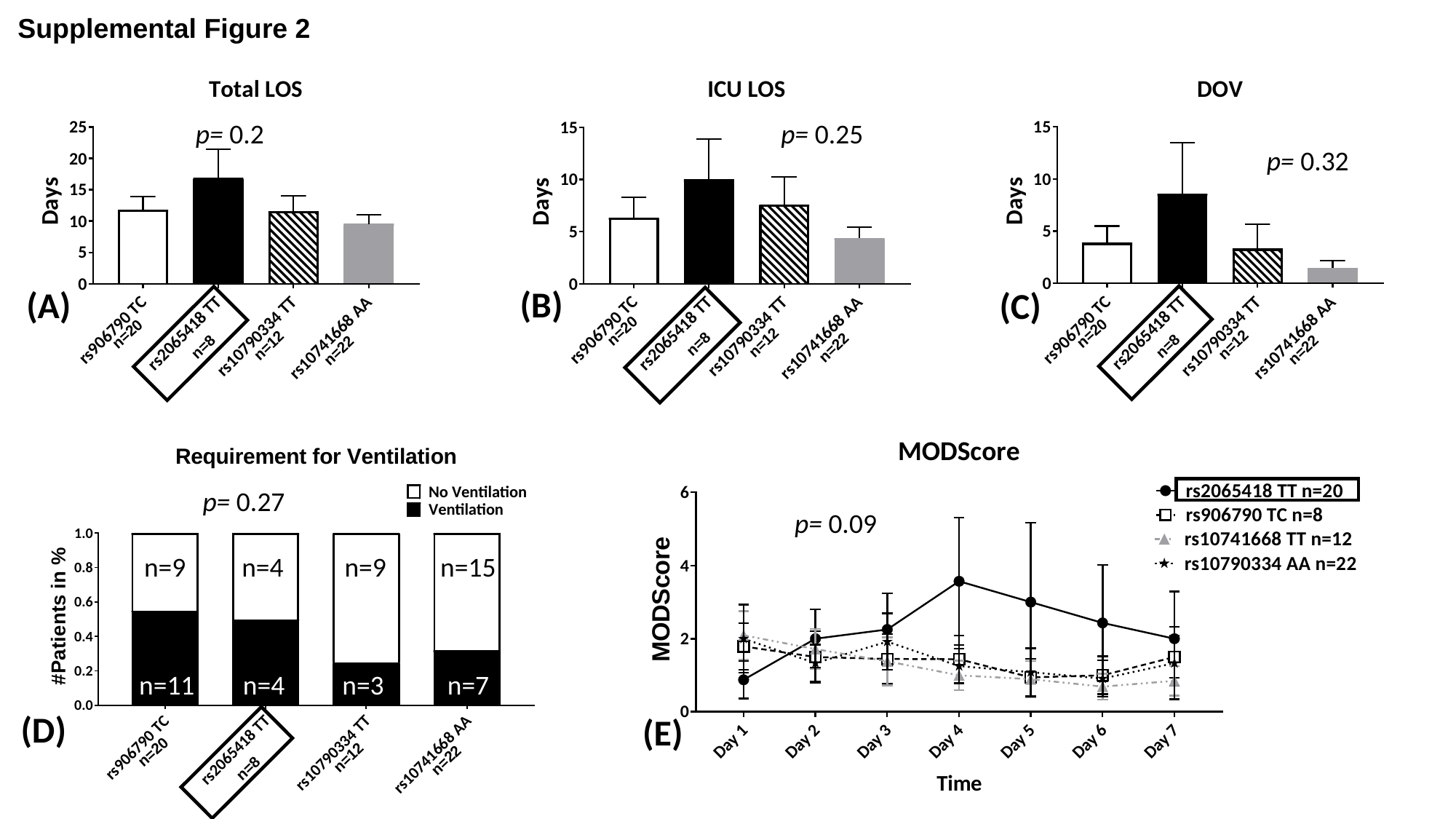

Supplemental Figure 2
p= 0.25
p= 0.2
p= 0.32
(B)
(A)
(C)
n=20
n=20
n=20
n=8
n=12
n=8
n=8
n=12
n=12
n=22
n=22
n=22
p= 0.27
p= 0.09
n=9
n=4
n=9
n=15
n=11
n=4
n=3
n=7
(D)
(E)
n=20
n=12
n=22
n=8

## Slide 3
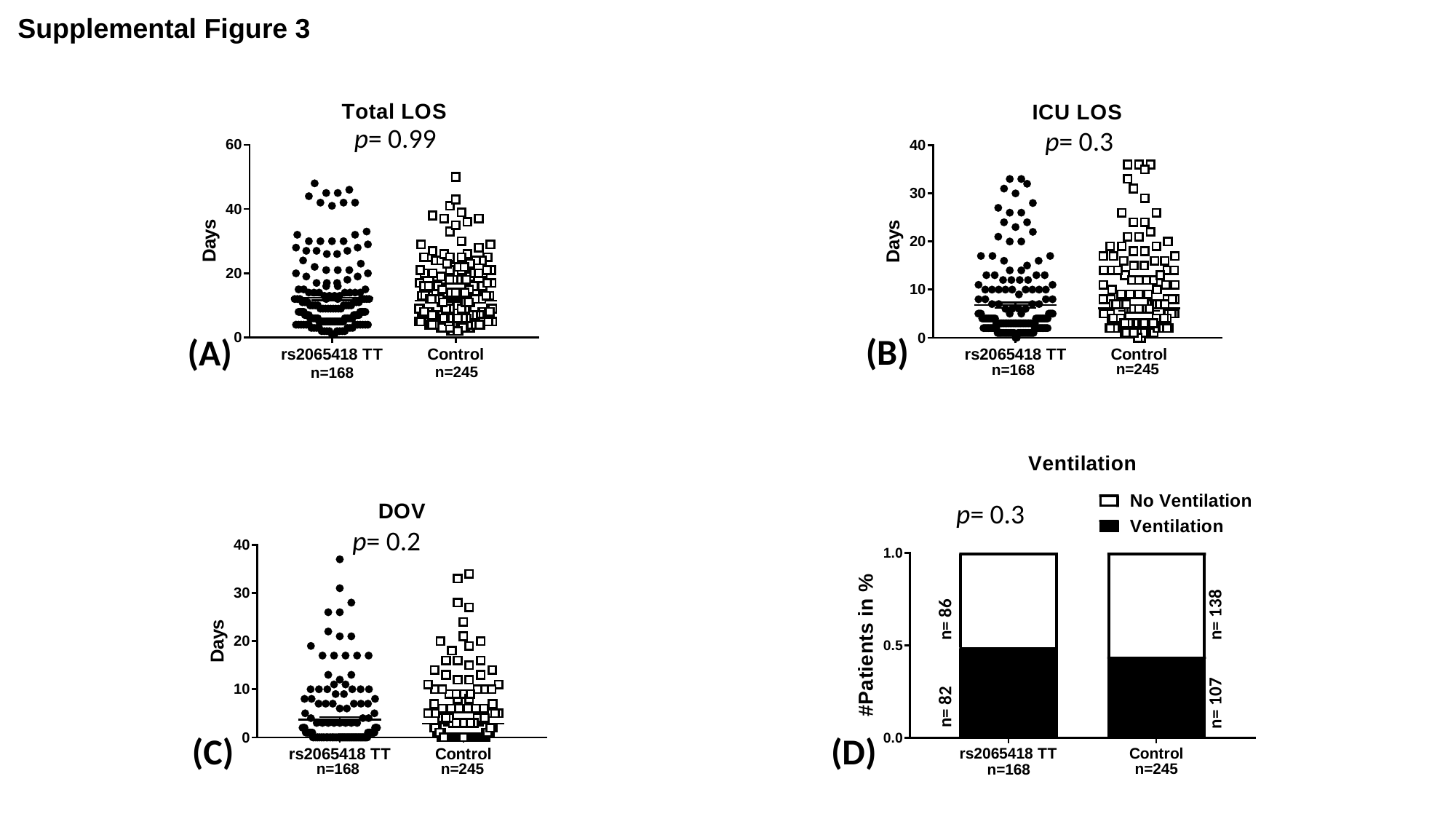

Supplemental Figure 3
p= 0.99
p= 0.3
(B)
(A)
n=245
n=168
n=245
n=168
p= 0.3
p= 0.2
n= 86
n= 138
n= 82
n= 107
(C)
(D)
n=245
n=245
n=168
n=168

## Slide 4
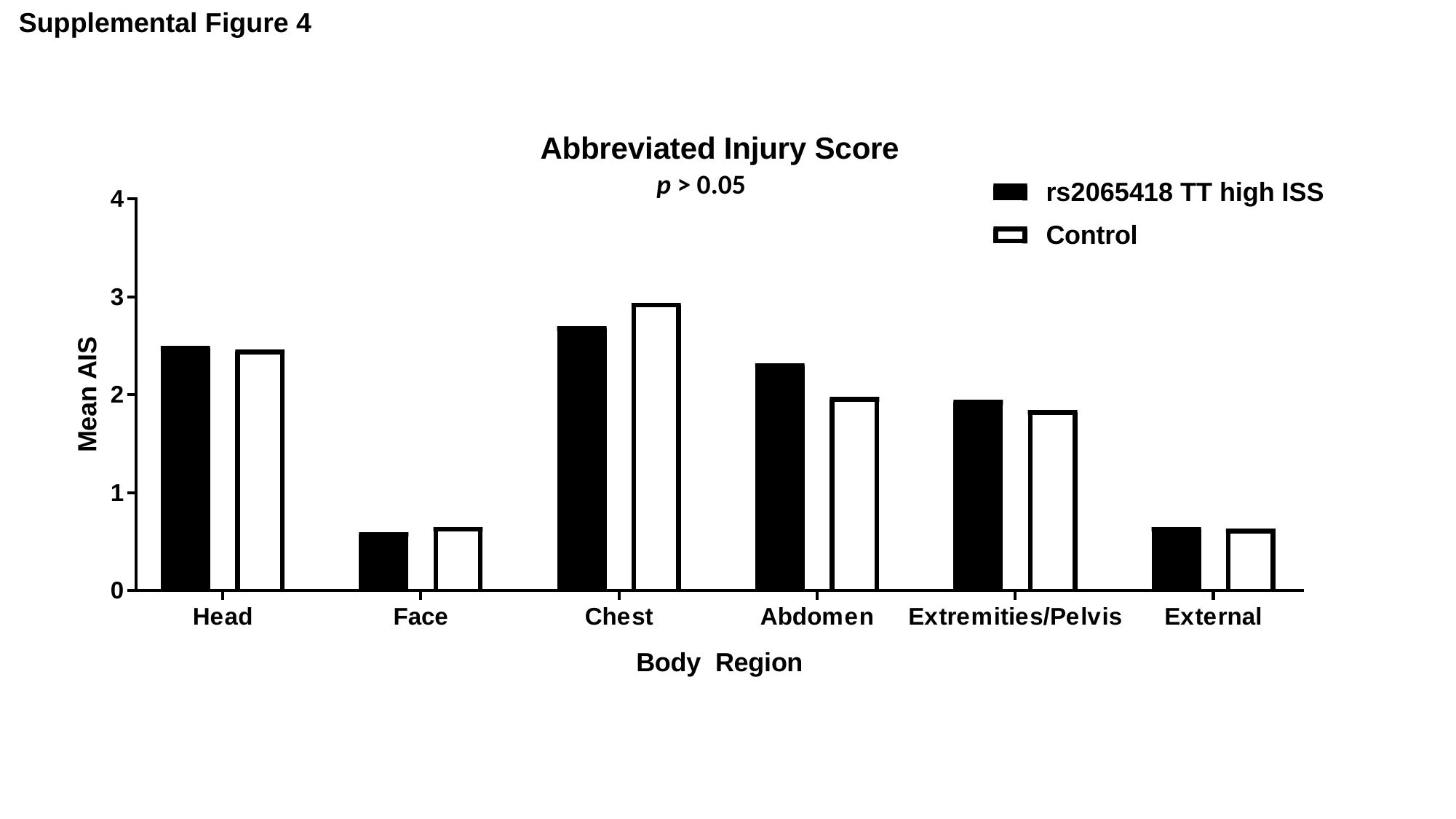

Supplemental Figure 4
p > 0.05

## Slide 5
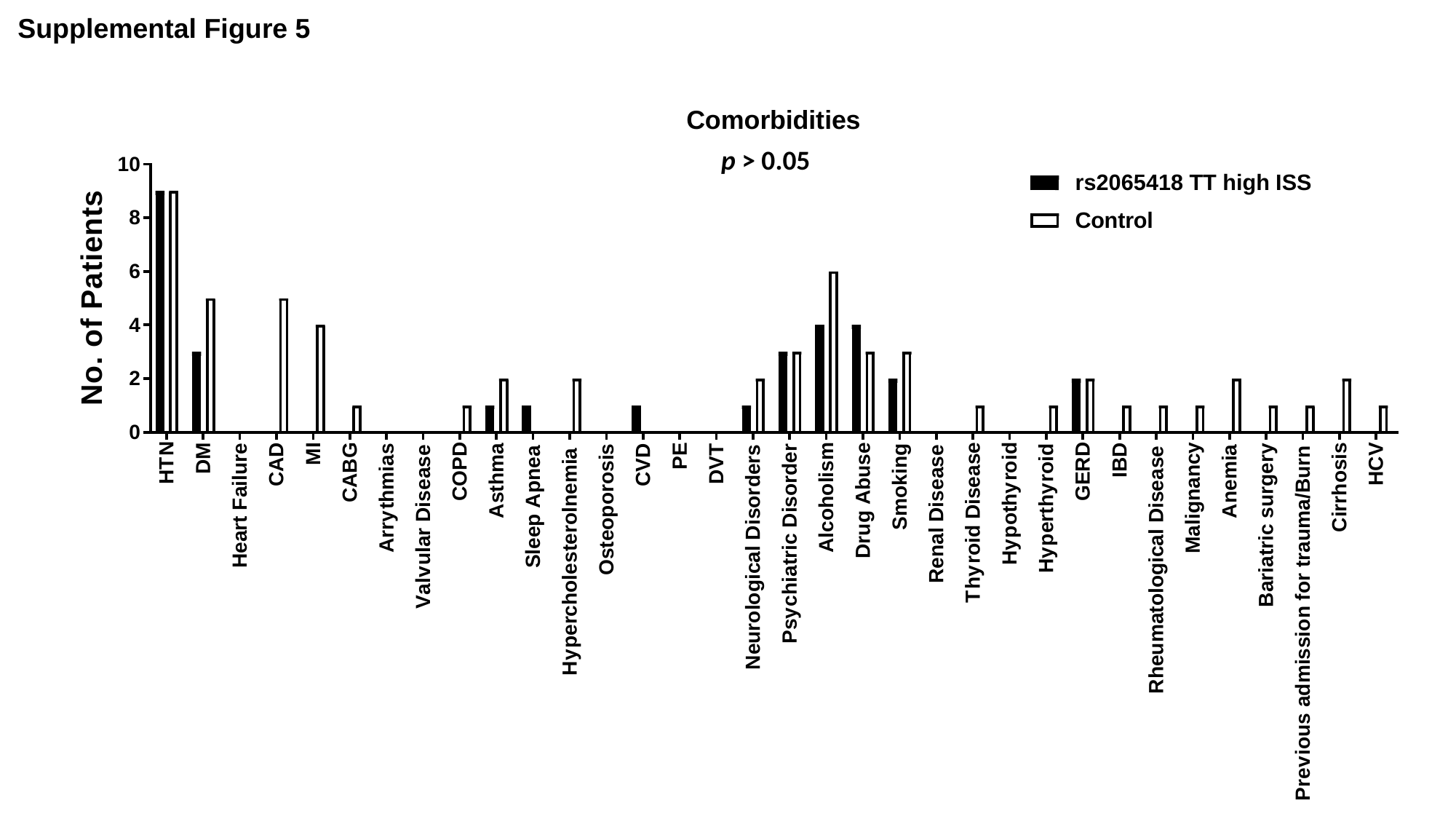

Supplemental Figure 5
p > 0.05

## Slide 6
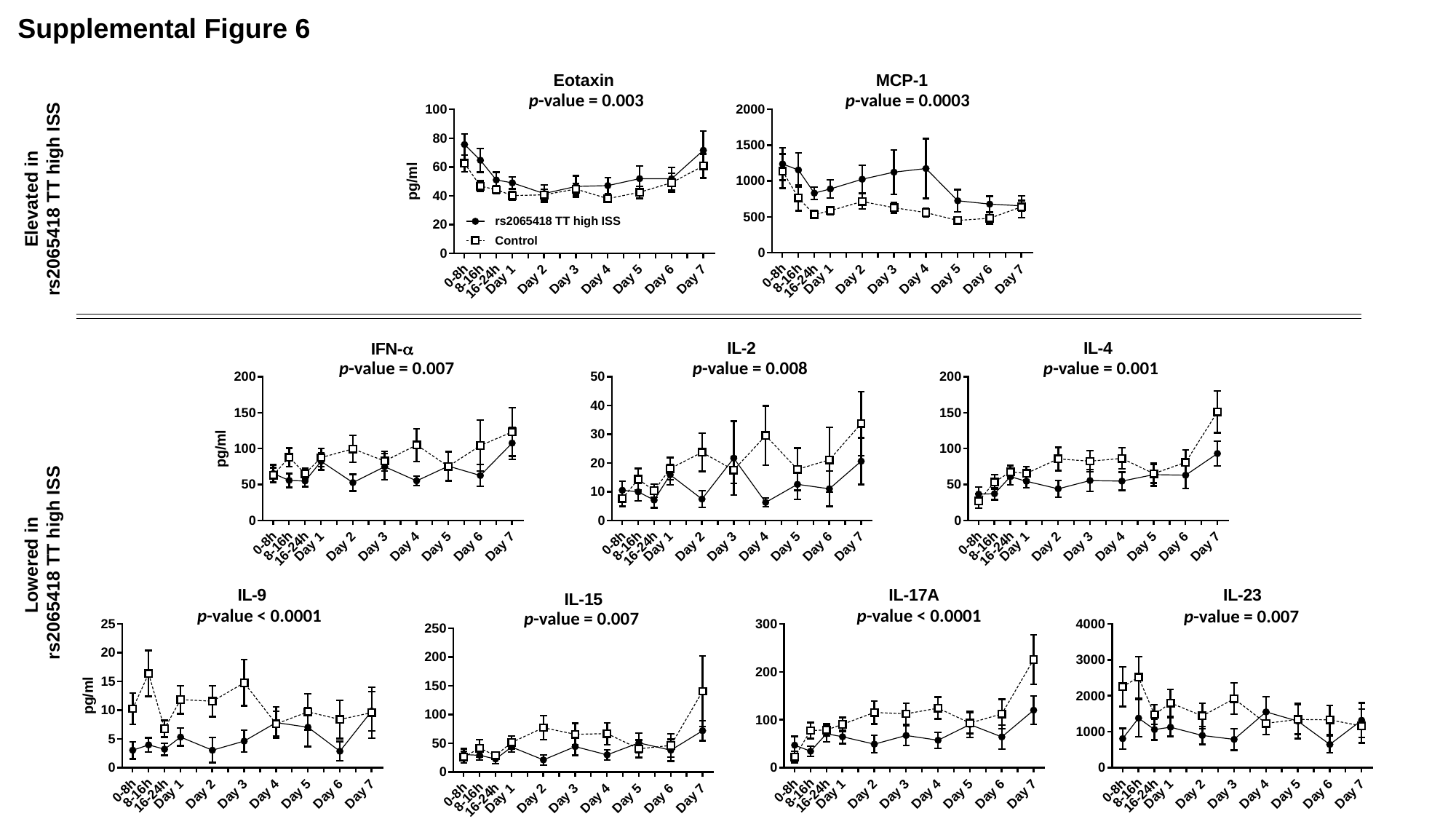

Supplemental Figure 6
p-value = 0.003
p-value = 0.0003
Elevated in
rs2065418 TT high ISS
p-value = 0.007
p-value = 0.001
p-value = 0.008
Lowered in
rs2065418 TT high ISS
p-value < 0.0001
p-value < 0.0001
p-value = 0.007
p-value = 0.007

## Slide 7
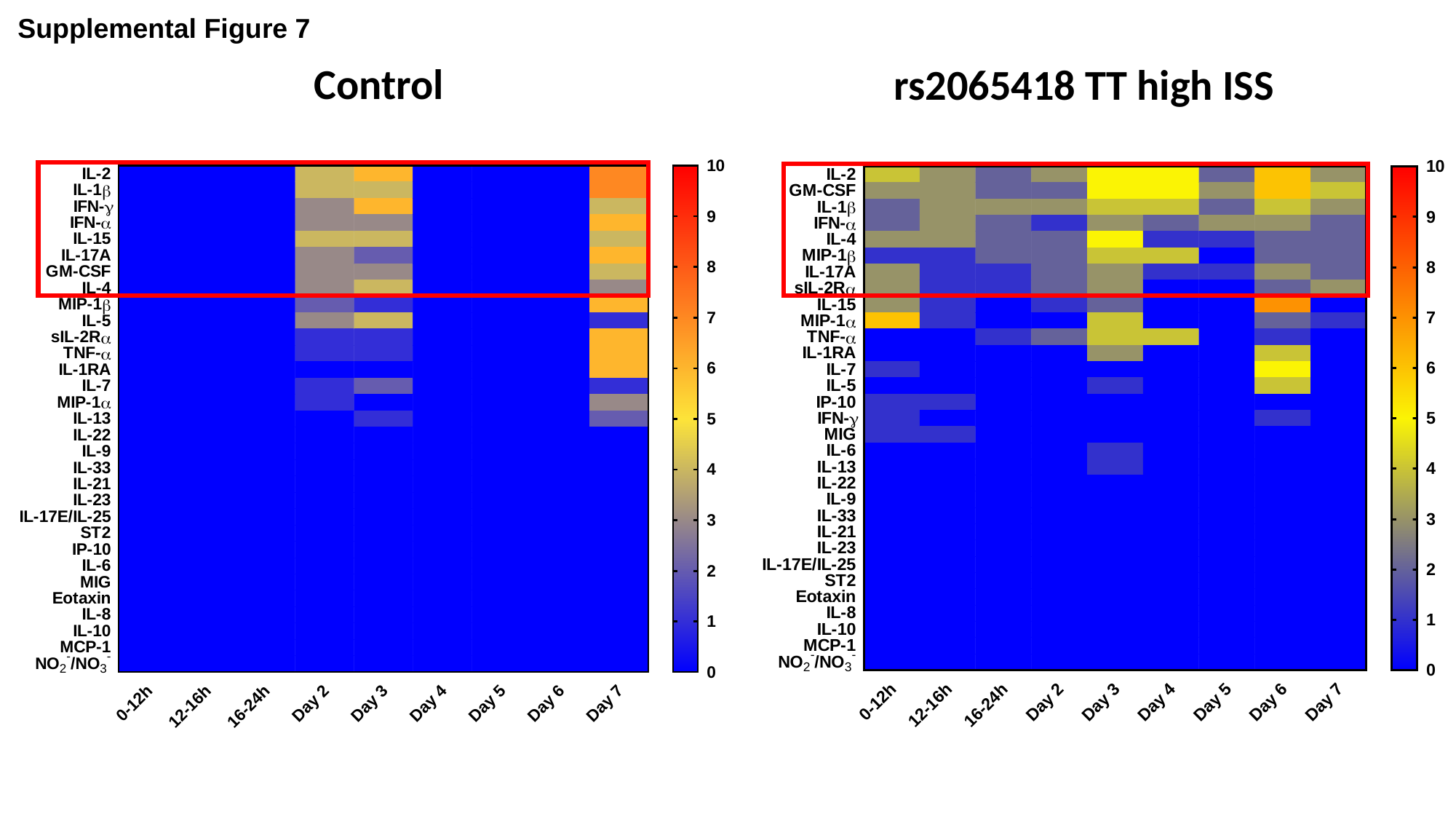

Supplemental Figure 7
Control
rs2065418 TT high ISS
