# Supplementary material for: MPPED2 Polymorphism Is Associated With Altered Systemic Inflammation and Adverse Trauma Outcomes
Source: Front Genet. 2019 Nov 8;10:1115. doi: 10.3389/fgene.2019.01115 (PMC6857553; doi:10.3389/fgene.2019.01115)
Supplement: Supplementary file 3 [file Presentation_2.pptx]

## Slide 1
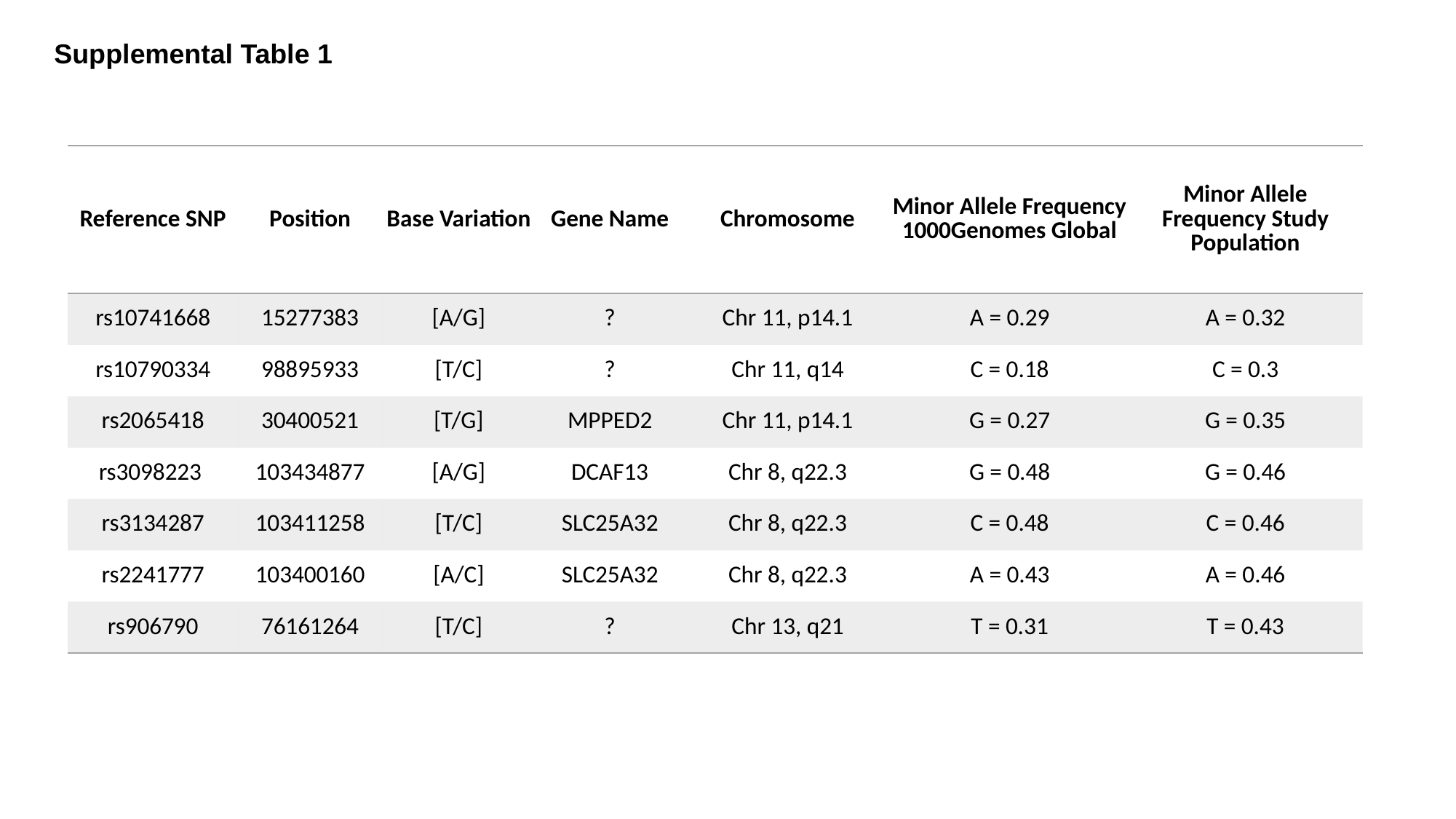

Supplemental Table 1
| Reference SNP | Position | Base Variation | Gene Name | Chromosome | Minor Allele Frequency 1000Genomes Global | Minor Allele Frequency Study Population |
| --- | --- | --- | --- | --- | --- | --- |
| rs10741668 | 15277383 | [A/G] | ? | Chr 11, p14.1 | A = 0.29 | A = 0.32 |
| rs10790334 | 98895933 | [T/C] | ? | Chr 11, q14 | C = 0.18 | C = 0.3 |
| rs2065418 | 30400521 | [T/G] | MPPED2 | Chr 11, p14.1 | G = 0.27 | G = 0.35 |
| rs3098223 | 103434877 | [A/G] | DCAF13 | Chr 8, q22.3 | G = 0.48 | G = 0.46 |
| rs3134287 | 103411258 | [T/C] | SLC25A32 | Chr 8, q22.3 | C = 0.48 | C = 0.46 |
| rs2241777 | 103400160 | [A/C] | SLC25A32 | Chr 8, q22.3 | A = 0.43 | A = 0.46 |
| rs906790 | 76161264 | [T/C] | ? | Chr 13, q21 | T = 0.31 | T = 0.43 |

## Slide 2
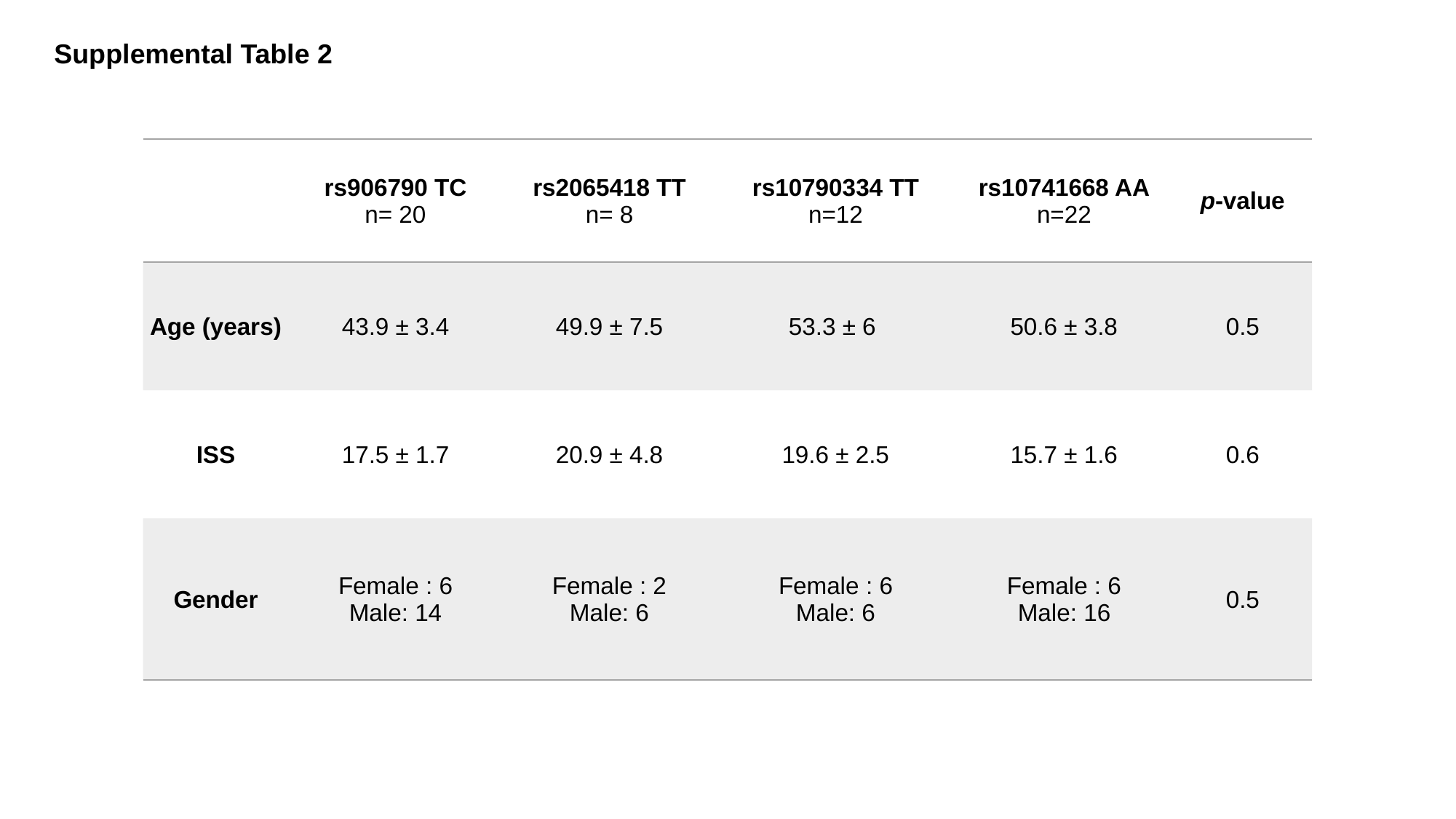

Supplemental Table 2
| | rs906790 TC n= 20 | rs2065418 TT n= 8 | rs10790334 TT n=12 | rs10741668 AA n=22 | p-value |
| --- | --- | --- | --- | --- | --- |
| Age (years) | 43.9 ± 3.4 | 49.9 ± 7.5 | 53.3 ± 6 | 50.6 ± 3.8 | 0.5 |
| ISS | 17.5 ± 1.7 | 20.9 ± 4.8 | 19.6 ± 2.5 | 15.7 ± 1.6 | 0.6 |
| Gender | Female : 6 Male: 14 | Female : 2 Male: 6 | Female : 6 Male: 6 | Female : 6 Male: 16 | 0.5 |

## Slide 3
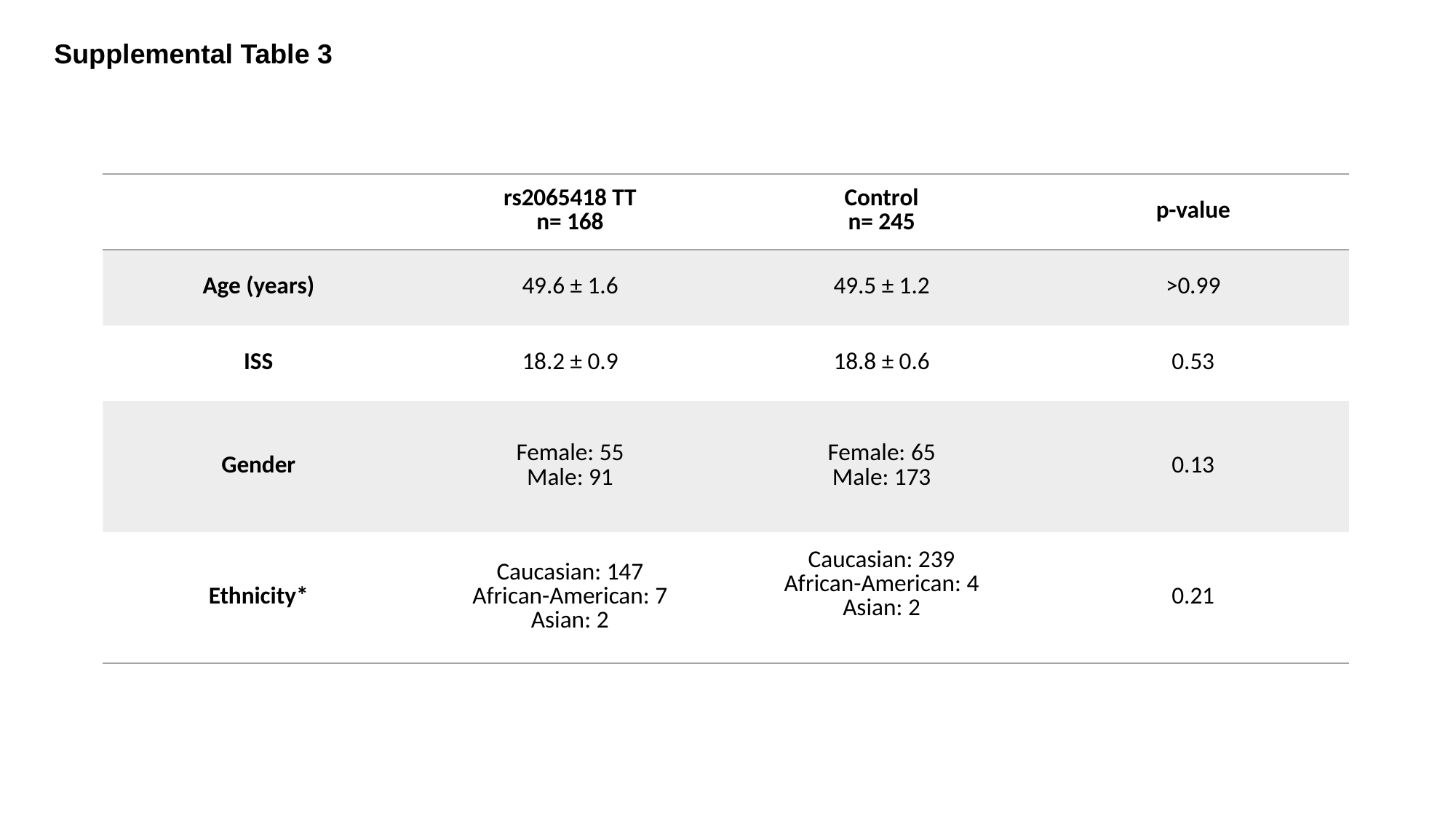

Supplemental Table 3
| | rs2065418 TT n= 168 | Control n= 245 | p-value |
| --- | --- | --- | --- |
| Age (years) | 49.6 ± 1.6 | 49.5 ± 1.2 | >0.99 |
| ISS | 18.2 ± 0.9 | 18.8 ± 0.6 | 0.53 |
| Gender | Female: 55 Male: 91 | Female: 65 Male: 173 | 0.13 |
| Ethnicity\* | Caucasian: 147 African-American: 7 Asian: 2 | Caucasian: 239 African-American: 4 Asian: 2 | 0.21 |

## Slide 4
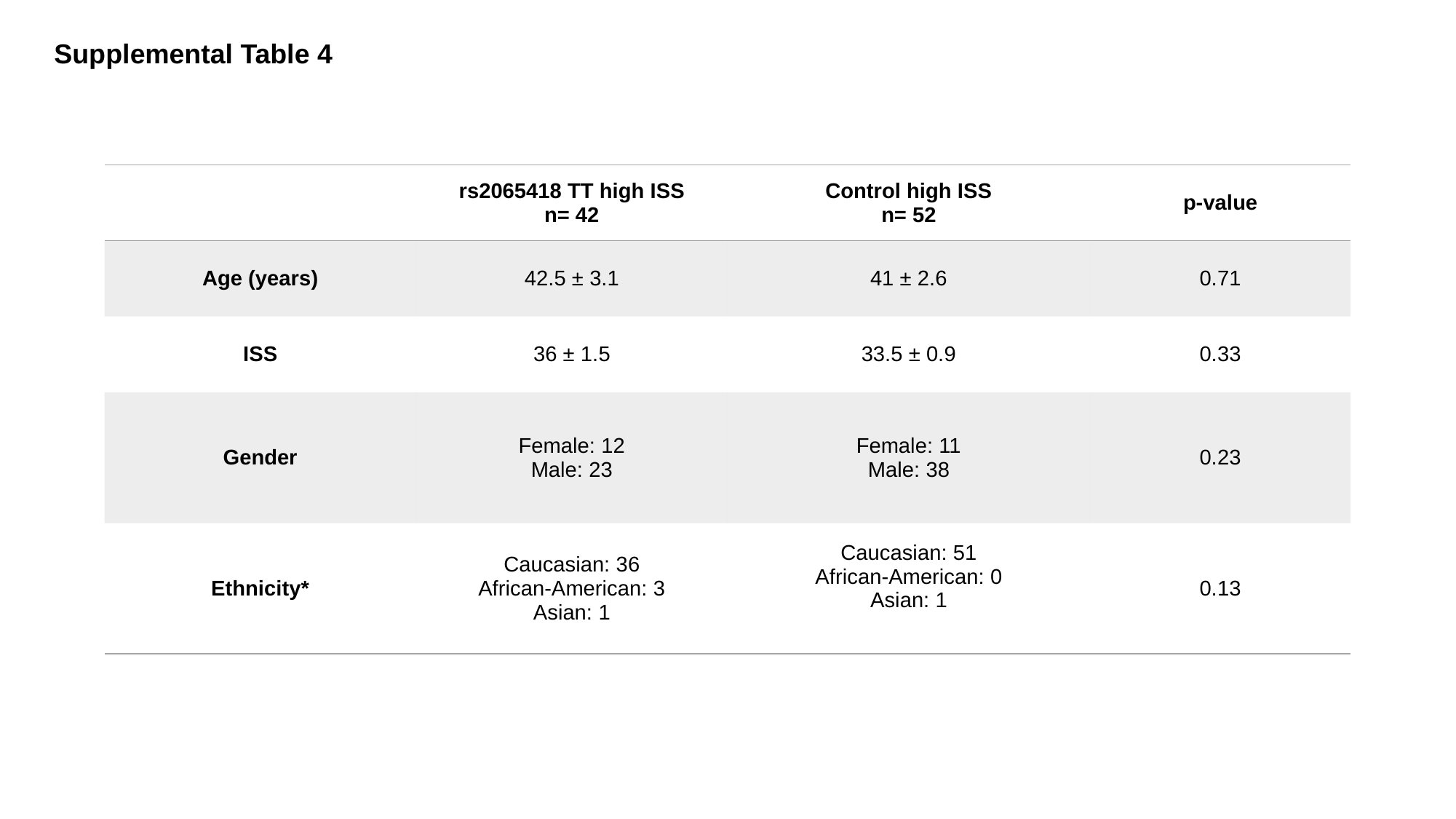

Supplemental Table 4
| | rs2065418 TT high ISS n= 42 | Control high ISS n= 52 | p-value |
| --- | --- | --- | --- |
| Age (years) | 42.5 ± 3.1 | 41 ± 2.6 | 0.71 |
| ISS | 36 ± 1.5 | 33.5 ± 0.9 | 0.33 |
| Gender | Female: 12 Male: 23 | Female: 11 Male: 38 | 0.23 |
| Ethnicity\* | Caucasian: 36 African-American: 3 Asian: 1 | Caucasian: 51 African-American: 0 Asian: 1 | 0.13 |

## Slide 5
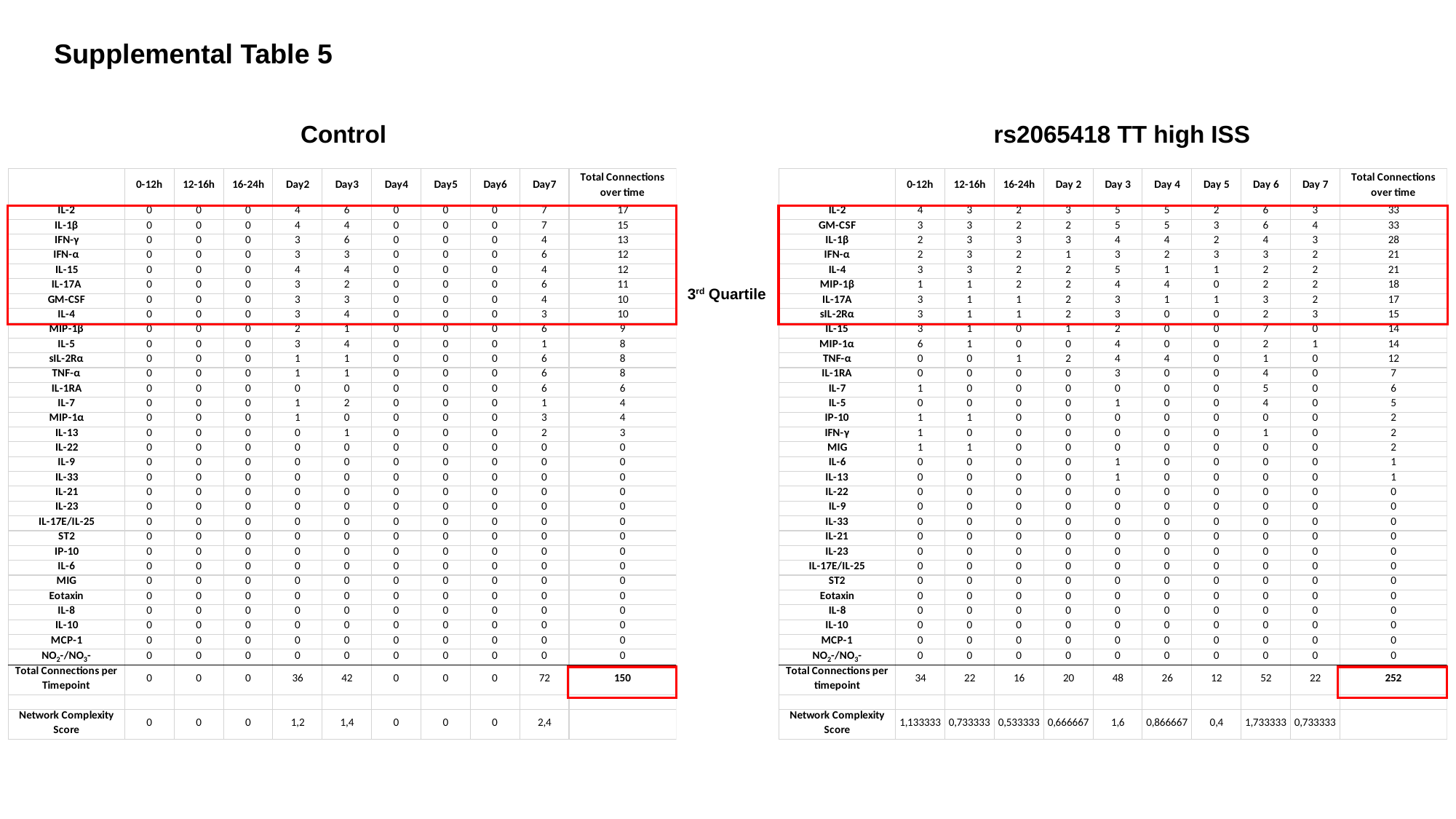

Supplemental Table 5
Control
rs2065418 TT high ISS
3rd Quartile
